# A Comparison of Artificial Intelligence and Human Doctors for the Purpose of Triage and Diagnosis

**DOI:** 10.3389/frai.2020.543405

**Published:** 2020-11-30

**Authors:** Adam Baker, Yura Perov, Katherine Middleton, Janie Baxter, Daniel Mullarkey, Davinder Sangar, Mobasher Butt, Arnold DoRosario, Saurabh Johri

**Affiliations:** ^1^ Babylon Health, London, United Kingdom; ^2^ Northeast Medical Group, Yale New Haven Health, New Haven, CT, United States

**Keywords:** virtual assistant, diagnosis, triage, diagnosis, AI diagnosis, symptom checker, computer-assisted diagnosis, bayesian networks

## Abstract

AI virtual assistants have significant potential to alleviate the pressure on overly burdened healthcare systems by enabling patients to self-assess their symptoms and to seek further care when appropriate. For these systems to make a meaningful contribution to healthcare globally, they must be trusted by patients and healthcare professionals alike, and service the needs of patients in diverse regions and segments of the population. We developed an AI virtual assistant which provides patients with triage and diagnostic information. Crucially, the system is based on a generative model, which allows for relatively straightforward re-parameterization to reflect local disease and risk factor burden in diverse regions and population segments. This is an appealing property, particularly when considering the potential of AI systems to improve the provision of healthcare on a global scale in many regions and for both developing and developed countries. We performed a prospective validation study of the accuracy and safety of the AI system and human doctors. Importantly, we assessed the accuracy and safety of both the AI and human doctors independently against identical clinical cases and, unlike previous studies, also accounted for the information gathering process of both agents. Overall, we found that the AI system is able to provide patients with triage and diagnostic information with a level of clinical accuracy and safety comparable to that of human doctors. Through this approach and study, we hope to start building trust in AI-powered systems by directly comparing their performance to human doctors, who do not always agree with each other on the cause of patients’ symptoms or the most appropriate triage recommendation.

## Introduction

AI virtual assistants (symptom checkers) are a convenient and valuable resource for users to better understand the underlying cause(s) of their symptoms and to receive advice on the most appropriate point of care (Millenson et al., 2018; Rowland et al., 2020). In low-income countries their impact may be even greater since, although access to human healthcare experts may be limited, many developing countries have seen a rapid expansion of mobile phones and wireless technology which has enabled opportunities to deliver mHealth technologies (Wahl et al., 2018). For example, a telehealth service that provides access to triage advice, GP appointments, prescriptions and tests is already used by two million patients in Rwanda and trials are underway to augment this service with AI technology (Burki, 2019).

Typically, symptom checkers cater to three healthcare needs of a patient. First is the provision of information, wherein a patient may seek to know more about the symptoms or conditions that they know or think they have. Secondly, a patient may want to know whether their symptoms require treatment or further investigation; this is medical triage and involves directing patients to the most suitable location within an appropriate time frame. The appropriate action depends on the nature and urgency of the symptoms or their underlying cause, which might require further investigation. Finally, patients may want to understand the conditions that might be responsible for their symptoms. This corresponds to diagnosis or “differential diagnosis” and is typically performed by an experienced medical practitioner.

Symptom checkers have the potential to alleviate the pressure on overly burdened healthcare systems and to reduce health disparities within under-served populations (Van Veen et al., 2019). For this to happen, healthcare professionals and the wider public must have confidence in the performance of symptom checkers and applications of AI to medicine more generally. Previous work has investigated the diagnostic and triage accuracy of symptom checkers (Semigran et al., 2015; Millenson et al., 2018; Chambers et al., 2019; Yu et al., 2019). In particular, (Semigran et al., 2015) assessed 23 commercial symptom checker apps and highlighted significant variation in terms of clinical accuracy. Whilst providing a useful benchmark, that study did not assess the accuracy of symptom checkers against the gold-standard performance of human doctors. This was assessed in a follow-up study, where the authors noted that doctors significantly outperform symptom checkers, providing a valuable contribution to our understanding of comparative diagnostic performance (Semigran et al., 2016). However, the method used in this follow-up study did not adequately assess the information gathering process through which patients typically interact with symptom checkers or doctors, and so the conclusions are not based on a fair or realistic comparison. Diagnostic accuracy is not routinely measured in clinical practice, but a wide range of studies have attempted to estimate the incidence of diagnostic error. Irrespective of whether the true error rate lies closer to the 10–20% found in autopsy studies (Graber, 2013) or the 44% found in a study analyzing the correlation of diagnostic accuracy with doctor confidence (Meyer et al., 2013), it is critical to perform a fair assessment of how a doctor takes a history and establishes a diagnosis when comparing against symptom checkers.

In this study we adopt a semi-naturalistic, role-play paradigm that simulates a realistic consultation between a patient and either our Triage and Diagnostic System or human doctor. Based on the assessment technique used throughout medical school and post-graduate medical qualifications (Objective Structured Clinical Examinations [OSCE]), this protocol was designed to assess not only the clinical (diagnostic^1^ and triage) accuracy, but also the ability to gather all of the relevant information from the patient, i.e., to take a history.

## Materials and Methods

### The Babylon Triage and Diagnostic System

The Babylon Triage and Diagnostic System is designed to provide users with triage advice alongside an explanation of why this action has been suggested; this consists of any reported symptoms that require urgent attention, and/or a list of possible causes for the user’s symptoms. A comprehensive description of the system that powers the Babylon Triage and Diagnostic System is outside of the scope of this paper, however we provide a brief summary of this system by way of background.

The Babylon Triage and Diagnostic System–a new implementation after the previous generation (Middleton et al., 2016)–is based on a Bayesian Network (Koller and Friedman, 2009) of primary care medicine, which models conditional dependencies between variables via a Directed Acyclic Graph (DAG). Nodes in the graph represent medical concepts and edges represent conditional dependencies. Here, we model medical concepts primarily as Boolean variables, where the states represent the presence or absence of that concept. A limited number of concepts are modeled as ordinal variables, for example symptom duration. Each node in the graph is associated with a Conditional Probability Table (CPT) which describes the state of the variable, given the states of its parents in the graph. We assume a three-layer DAG structure, where the top, middle and bottom layers represents risk factors, disease and symptoms, respectively. Edges may exist between risk factors and diseases or between diseases and symptoms. One challenge when creating large scale Bayesian Networks is that the number of parameters required to specify the CPT is exponential in the number of parent nodes. This can make parameterization of the CPT challenging, and subsequent inference intractable. We adopt a noisy-OR model, which is widely used in diagnostic modeling as it greatly simplifies inference, allowing large networks to be described by a number of parameters that grows linearly with the size of the network (Heckerman, 1990). Under the noisy-OR assumption, diseases cause symptoms independently of other diseases, and the CPT for a symptom when multiple diseases are present may be derived through a logical OR function of its parents. The model is parameterized by the prior probabilities of risk factors and diseases, and the conditional probabilities which define the CPTs between risk factors and diseases and between diseases and symptoms. Prior probabilities are obtained from epidemiological data which varies to reflect local disease and risk factor burden in different regions and population segments. Sources of epidemiological data include published literature and data from the Global Burden of Disease (GBD) study, which provides age- and sex-stratified estimates of disease incidence and prevalence in different regions^2^. The range of disease incidence values used to calculate the priors in our model are shown in Supplementary Table S1. The GBD dataset was augmented with data from literature searches by our in-house epidemiology team, and estimates from medical experts. Conditional probabilities (for example, the probability of a symptom occurring given a disease) were obtained through elicitation from multiple independent medical experts.

Once constructed and parameterized, the model may be used to reason about the possible underlying disease or diseases that explain the user-entered symptoms and risk factors, using a variety of Bayesian inference methods (Cheng and Druzdzel, 2000; Wainwright and Jordan, 2008; Gu et al., 2015; Douglas et al., 2017). This allows the AI powered Triage and Diagnostic System to output the most likely causes of the symptoms entered by a user, and also generate follow up questions that provide the most information to confirm or rule out the diseases under consideration. It should be noted that while we use posterior inference in this study (i.e., the application of approximate Bayesian inference to calculate the posterior probabilities given a prior belief and evidence), the same model structure may be used in conjunction with other inference schemes such as counterfactual inference.

The Babylon Triage and Diagnostic System is designed to identify one of six mutually-exclusive triage recommendations: “call an ambulance”, “go to A&E/ER”, “urgent GP” (i.e., within 6 h), “non-urgent GP” (i.e., within a week), “pharmacy” and “self-care”. These are ordered in terms of urgency from highest to lowest. We define under-triage and over-triage to be recommendations from the system that are of a lower or higher urgency than is appropriate. The triage capability is based on a utility model, which provides a generalization of the Bayesian Network to a decision network (Koller and Friedman, 2009). This combines the likelihood of each disease with the potential harm caused by that disease, under all possible triage decisions. The triage decision that is recommended is the one that minimizes the expected harm to the patient, while also penalizing over-triaging. This model calculates the expected harm (i.e., negative expected utility) associated with each disease as the probability of that disease multiplied by the harm that would be incurred by taking a particular triage decision, should that disease exist. The total expected harm to the patient for each triage decision is calculated as the sum of expected harm for each possible disease. The total expected utility for a triage decision is the expected harm, plus a separate cost term which depends only on the triage decision. The effect of this cost term is to penalize over-triaging, by making urgent triage decisions more costly. The utility model is parameterized using distributions over clinical outcomes for each disease (e.g., the proportion of patients with the disease that would experience a particular clinical outcome). The costs associated with these outcomes, and the costs for each triage decision are learned from a combination of simulated and real clinical cases (which were distinct from those used within this study). To guarantee the safe triage of patients with symptoms that require a particular urgency or location of care (regardless of their underlying cause), the utility model is augmented with a set of rules that dictate a specific triage action where a particular combination of symptoms (so-called “red-flag” symptoms) are present.

### Experimental Paradigm

We compared the accuracy and safety of the Babylon Triage and Diagnostic System against that of human doctors. Accuracy was assessed in terms of the relevance of the suggested conditions, and the appropriateness of the recommended triage action. Triage safety was assessed in terms of whether the suggested triage action was deemed safe (even if it was overly cautious).

**FIGURE 1:**
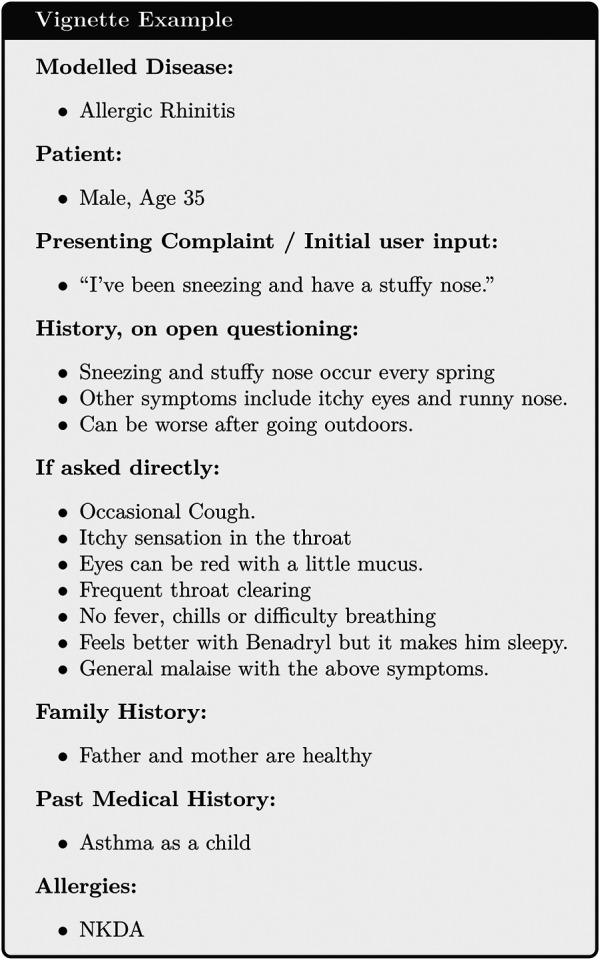
An example of a test vignette.

The evaluation was performed using a semi-naturalistic role-play scenario that involved mock consultations between a patient and either a human doctor or the chatbot, based on realistic clinical vignettes. The role of doctors was played by general practitioners (GPs) who were hired on a locum basis for the experiment and who were not involved with the development of the model. Patients were played by GPs, some of whom were employees of Babylon, but none of whom were involved with the development of the model. We opted to use GPs to play the patients instead of professional actors as in a previous study (Middleton et al., 2016) to prioritize the accuracy of interpreting the information on the vignette over the realism of a layperson. One hundred clinical vignettes were created by independent medical practitioners who were not involved in the role-play experiment (see Supplementary Table S2 for further details of the participants of the study). Each vignette was designed to simulate a medical condition from the list of all conditions currently modeled by the Triage and Diagnostic System^3^, in a patient of at least 16 years of age. The vignettes contained information about the patient, their initial complaint(s), information about their symptoms and past medical history that should be offered on open questioning, and information that should only be reported on direct questioning. An example can be found in Figure 1.

The study was conducted in June 2018 and consisted of four rounds over consecutive days. In each round, there were up to four “patients” and four doctors. Each patient was assigned a vignette as their presenting history and had independent consultations with each doctor and the Babylon Triage and Diagnostic System. This protocol was designed in the OSCE format to assess both history taking and diagnostic and triage accuracy. After each consultation the differential diagnosis and recommended triage produced by the doctor or Triage and Diagnostic System was recorded. In order to maintain blinding in the judging process, doctors selected their differential diagnoses from a list of all conditions covered by the Triage and Diagnostic System. Once the patient had completed consultations with all doctors and the Triage and Diagnostic System, they were assigned a new vignette and the process was repeated.

## Results

### Accuracy of Differential Diagnosis Against Vignette Modeled Disease

We assessed the precision (also called positive predictive value) and recall (also called sensitivity) of the Babylon Triage and Diagnostic System and doctors against the condition on which the vignette was based. Recall is the proportion of relevant diseases that are included in the differential. When considering only the single disease modeled by the vignette, this corresponds to the proportion of differentials that contained this disease, over all vignettes. Precision is the proportion of the diseases in the differential that are relevant. A precision of one hundred percent would be achieved if the differential diagnosis contained only the disease modeled by the vignette. In general this level of certainty is unlikely and even undesirable, given only the information provided on the vignette (i.e. in the absence of diagnostic tests), but penalizes overly long differentials that would result in a higher recall.

In this study, the Babylon Triage and Diagnostic System was able to produce differential diagnoses with precision and recall comparable to that of doctors, and in some cases exceeded human level performance (Table 1). The average recall of doctors was found to be 83.9%, (64.1–93.8%), meaning that doctors failed to include the vignette disease in their differential in sixteen percent of cases on average.

**TABLE 1:** Diagnostic performance for all seven doctors and the Babylon Triage and Diagnostic System (Babylon AI).

	Average recall (%)(95% CI)	Average precision (%)(95% CI)	F1-score (%)(95% CI)	Number of vignettes
Doctor A	80.9	42.9	56.1	47
Doctor B	64.1	36.8	46.7	78
Doctor C	93.8	53.5	68.1	48
Doctor D	84.3	38.1	52.5	51
Doctor E	90.0	33.9	49.2	70
Doctor F	90.2	43.3	58.5	51
Doctor G	84.3	56.5	67.7	51
Doctor average	**83.9**	43.6	57.0	56.6
—	(75.6–92.3)	(36.3–50.9)	(49.7–64.2)	—
Babylon AI	80.0	**44.4**	**57.1**	100

The diagnostic performance of the Babylon Triage and Diagnostic System is comparable to that of doctors in terms of the recall, precision (positive predictive value) and F1-score (harmonic mean of precision and recall) against the disease modeled by the clinical vignette.

**FIGURE 2:**
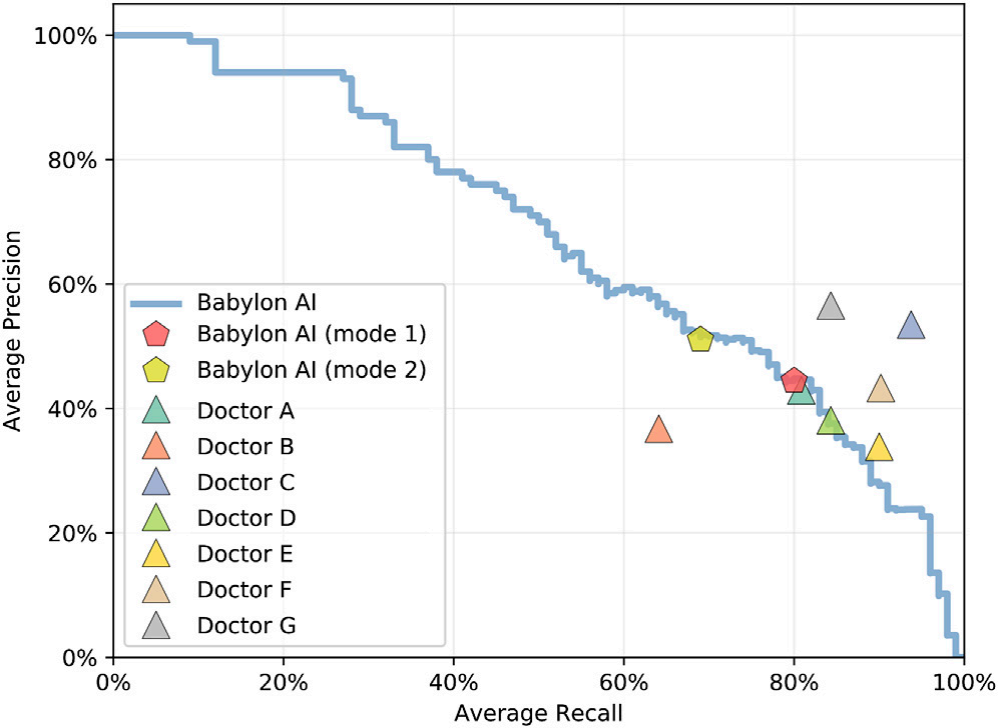
Average recall and precision for doctors and for the Babylon Triage and Diagnostic System (Babylon AI) for different threshold parameters, showing the operating point typically used (mode 1) and an alternative operating point (mode 2) that favors higher precision at the expense of reduced recall. Varying the internal thresholds allows the model to behave more similarly to different individual doctors, while maintaining a high level of performance, suggesting that it is not overly tuned to a particular operating point.

The Babylon Symptom Selector is based on a Bayesian model, which can calculate the posterior probabilities of all conditions in the model given the evidence known about a patient. Whether particular conditions are displayed to the user depends on whether they meet internal thresholds, defined by a combination of the probability and severity of these conditions. The threshold parameters used in the model are selected based on independent training vignettes but may be varied to allow a trade-off to be made between recall and precision. It is interesting to observe that different parameters can move the model’s result closer to those of different doctors, for example toward Doctor D or E (Figure 2), perhaps emulating the variability in individual doctors’ preference for shorter, more precise differentials or longer, more exhaustive ones.

### Expert Rating of Differential Diagnoses

In addition to assessing the precision and recall compared to the disease modeled by the vignette, we also evaluated the overall differential diagnosis qualitatively. This was based on the intuition that, to be useful, a differential diagnosis must not only include the relevant diseases but also exclude diseases that are of little relevance to the patient’s symptoms. To this end, we asked a senior medical practitioner^4^ (Judge 1) and two in-house GPs (Judge 2 and Judge 3) who were not part of the role play experiment, to serve as judges and to independently rate the quality of the differentials produced both by the Babylon Triage and Diagnostic System and by doctors. Each judge first reviewed the vignette and then rated all the differentials for this vignette on a four point scale (poor, okay, good, excellent). A differential was rated “excellent” if the judge could not find any issues with it, “good” if it had minor issues (such as the omission of a slightly irrelevant conditions, or if the order of the differential was deemed imperfect), “okay” if the list of conditions was generally acceptable, and “poor” if it was unacceptable (such as the omission of the most important conditions, or the inclusion of diseases completely unrelated to the presenting symptoms). The differentials were shown in random order and the judge was blinded to whether the differential had been produced by a human or the Babylon Triage and Diagnostic System.

**FIGURE 3:**
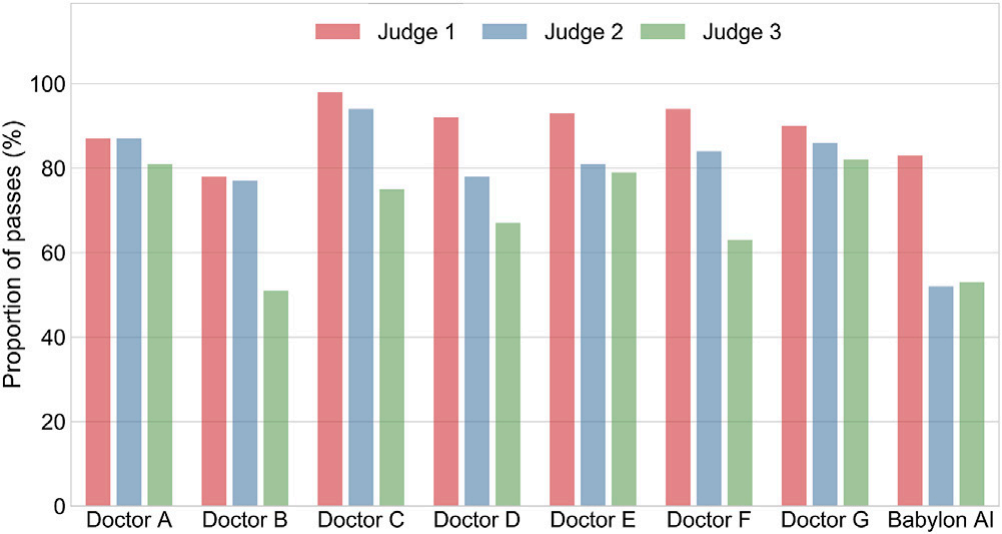
Percentage of differential diagnoses rated as “okay” or better by three judges for doctors and the Babylon Triage and Diagnostic System (Babylon AI). There is considerable disagreement between the three ratings, suggesting the qualitative assessment of differential diagnoses might be influenced by personal preference.

We found that there was considerable disagreement between the medical practitioners’ subjective assessment of the differentials (see Figure 3; Supplementary Tables S3–S5). For Judge 1, the lists of diseases output by the Babylon Triage and Diagnostic System were found to be of comparable quality to those produced by doctors (83.0% rated “okay” or better, compared to 78.2–97.9%). The same was the case for one of the GPs (Judge 3), who was generally harsher on the evaluation (53.0% rated “okay” or better, compared to 51.3–82.4%). However, another GP (Judge 2) rated the quality of differentials of the Babylon Triage and Diagnostic System lower than those of doctors (52.0% rated “okay” or better, compared to 76.9–93.8%).

**FIGURE 4:**
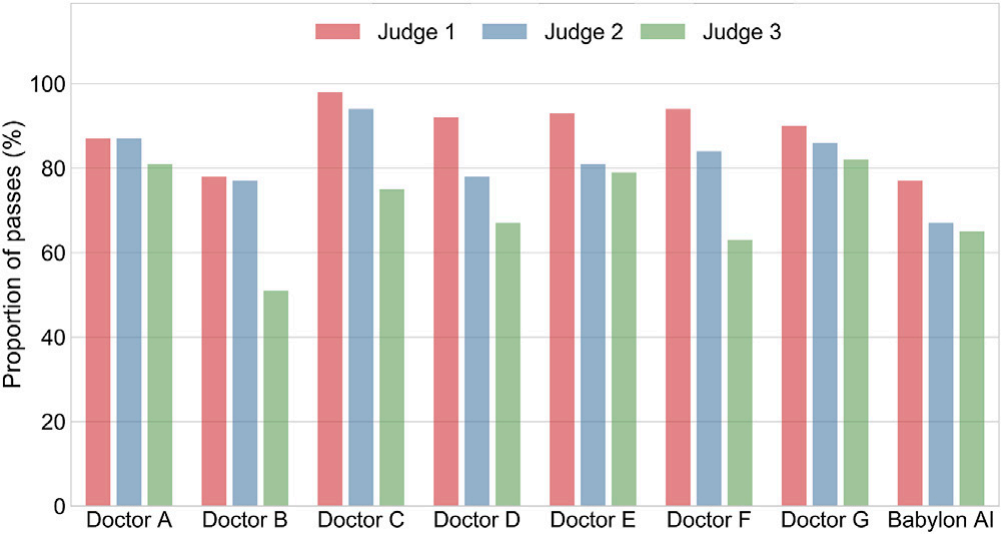
Percentage of differential diagnoses rated as “okay” or better by three judges for doctors and the Babylon Triage and Diagnostic System (Babylon AI), where the latter is tuned to provide higher precision at the expense of recall (mode 2). The differentials provided by the Babylon Triage and Diagnostic System were rated to be of comparable quality to those of doctors.

We considered that the disparity in the qualitative evaluation of differential diagnoses might be due to a difference in interpretation and that some medical practitioners might be less tolerant of disease lists that are long or contain less relevant diseases, even if the relevant conditions are included. We repeated the experiment with the Babylon Triage and Diagnostic System tuned to provide higher precision at the expense of lower recall (Figure 2; mode 2). This mode resulted in a marked improvement in the ratings of the GPs, (Judges 2 and 3) which may suggest a preference for more concise differentials for these individuals (Figure 4).

### Assessment of Triage Safety and Appropriateness

In addition to rating the quality of doctors differential diagnoses, the expert judge (Judge 1) was also asked to specify a range of safe and appropriate triage outcomes for each vignette. Providing a range of acceptable triage recommendations was motivated by the fact that doctors often disagree on the most appropriate triage recommendation (Supplementary Figure S1), however it is not necessarily the case that any of these different opinions are inappropriate or unsafe (O’Cathain et al., 2003). By providing the minimum and maximum appropriate triage, our judge indicates the range of recommendations that are neither unsafe nor overly cautious.

We compared the triage recommendations of doctors and the Babylon Triage and Diagnostic System against the judge’s “gold standard” range. We define a “safe” triage as any recommendation which was of equal or greater urgency than the judge’s minimum triage, and an “appropriate” triage as any recommendation that fell within the judge’s range of acceptable recommendations. In this study, we found that the Babylon Triage and Diagnostic System provided a safer triage recommendation than doctors on average (97.0% vs. 93.1%), at the expense of a marginally lower appropriateness (90.0% vs. 90.5%; see Table 2).

**TABLE 2:** Safety and appropriateness of triage recommendations for doctors and the Babylon Triage and Diagnostic System (Babylon AI) against a range of acceptable recommendations provided by an independent judge.

	Safety(95% CI)	Appropriateness(95% CI)	Number of vignettes
Doctor A	95.7	91.5	47
Doctor B	89.7	89.7	78
Doctor C	100.0	93.8	48
Doctor D	94.1	94.1	51
Doctor E	90.0	85.7	70
Doctor F	94.1	90.2	51
Doctor G	88.2	88.2	51
Doctor average	93.1	**90.5**	56.6
—	(89.6–96.6)	(87.9–93.0)	—
Babylon AI	**97.0**	90.0	100

The Babylon Triage and Diagnostic System gives safer triage recommendations than the doctors on average, at the expense of a marginally lower appropriateness.

We repeated this process with three in-house GPs and found the triage safety and appropriateness of the Babylon Triage and Diagnostic System relative to the doctors to be consistent with those of the judge, although the scores from the GPs were found to be lower for both the Babylon Triage and Diagnostic System and the doctors (Table 3).

**TABLE 3:** Safety and appropriateness of triage recommendations for doctors and the Babylon Triage and Diagnostic System (Babylon AI) against a range of acceptable recommendations provided by GPs.

	GP-1		GP-2		GP-3	
	Safety (%)	Appr. (%)	Safety (%)	Appr. (%)	Safety (%)	Appr. (%)
	(95% CI)		(95% CI)		(95% CI)	
Doctor A	97.9	89.4	91.5	83.0	95.7	89.4
Doctor B	79.5	75.6	60.3	59.0	75.6	74.4
Doctor C	97.9	89.6	93.8	89.6	95.8	93.8
Doctor D	80.4	76.5	64.7	62.8	86.3	84.3
Doctor E	84.3	78.6	70.0	67.1	80.0	78.6
Doctor F	92.2	86.3	74.5	68.6	92.2	84.3
Doctor G	92.2	88.2	72.6	70.6	84.3	80.4
Doctor average	89.2	**83.5**	75.3	71.5	87.1	**83.6**
—	(82.5–95.9)	(78.1–88.8)	(64.4–86.3)	(62.1–80.9)	(80.4–93.8)	(78.0–89.2)
Babylon AI	**90.0**	74.0	**81.0**	**75.0**	**90.0**	81.0

The AI powered System gives safer triage recommendations than the doctors on average, at the expense of a slightly lower appropriateness.

### Performance Against Publicly Available Case Vignettes

In order to provide a benchmark against previous work, as well as to the diagnostic accuracy that is expected for human practitioners, we assessed the performance of the Babylon Triage and Diagnostic System against the set of publicly available case vignettes used in a previous study.

The methodology described previously was repeated for 30 vignettes from a previous study by (Semigran et al., 2015). Following the methodology of the original study, we excluded vignettes that were based on conditions that fall outside of the scope of the Babylon Triage and Diagnostic System. Specifically, these included pediatric and dermatological conditions, and tetanus which is not currently in the model yet based on its very low incidence rate in the United Kingdom. These vignettes were tested against both the Babylon Triage and Diagnostic System and three doctors. As per the original study, we report the recall of the condition modeled by the vignette for the top one and top three conditions listed in the differential. The Babylon Triage and Diagnostic System identified the modeled condition as its top one in 21 out of 30 vignettes (70.0%) and in its top three in 29 out of 30 vignettes (96.7%). On average, doctors identified the modeled condition in their top one in 75.3% of vignettes and in their top three in 90.3% of vignettes. This demonstrates a considerable improvement relative to other symptom checkers evaluated in the original study.

## Discussion

We performed a prospective validation study of the accuracy and safety of an AI powered Triage and Diagnostic System, using an experimental paradigm designed to simulate realistic consultations. Overall we found that the Babylon Triage and Diagnostic System was able to identify the condition modeled by a clinical vignette with accuracy comparable to human doctors (in terms of precision and recall). We also found that the triage advice recommended by the Babylon Triage and Diagnostic System was safer on average than human doctors, when compared to the ranges provided by independent expert judges, with only minimal reduction in appropriateness. In other words, the AI system was able to safely triage patients without reverting to overly pessimistic fallback decisions.

We adopted a test protocol using simulated clinical vignettes which allowed us to evaluate a combination of common and rare conditions, the latter of which would be difficult to evaluate without a clinical trial with a sample size large enough to contain diseases with low incidence rates. While this might be considered a strength of our study, since it is not biased toward common presentations, our results cannot be directly interpreted with respect to real-world accuracy and safety. To illustrate the differences that might be expected in a real-world study, we reweighted our results by the annual incidence of the modeled disease for each vignette. We found that the accuracy and rating of differentials produced by the Babylon Triage and Diagnostic System improved compared to those of doctors after accounting for disease incidence (Supplementary Table S6 and Supplementary Figure S2). Surprisingly, we found that the accuracy and rating of some doctors decreased considerably after reweighting. This is likely due to the fact that the most common conditions carry substantially more weight than the rarer ones; thus the results will be highly sensitive to a few vignettes (in particular, Doctor A did not include a modeled disease in their differential for a vignette, where that modeled disease was very common and hence had high weight). Further work will be required to more rigorously investigate the diagnostic accuracy in a real-world clinical setting.

One source of bias in this study derives from the limitation imposed on doctors to only select diseases that are modeled in the Babylon Triage and Diagnostic System. As the “correct” disease for each vignette was always from this list, this may have provided human doctors with some advantage in terms of precision and recall compared to free text entry. However, it would have also constrained them from providing a fuller and more nuanced differential diagnosis overall, which may have disadvantaged them in terms of judge rating of overall differential quality. The intention in assigning this limitation as part of the testing protocol was to ensure blinding when the judges assessed the quality of the differential diagnosis.

Another possible limitation of our study is that we evaluated only clinical cases that were based on a single underlying condition (although we did include past medical history and pre-existing conditions). In reality, patients may have multiple undiagnosed diseases. However, one of the strengths of our approach, which uses a Bayesian model, is that it is able to reason about multiple causes of a patient’s presenting symptoms. It would be useful to test whether the performance relative to doctors is different in cases where multiple diseases must be diagnosed.

This study emphasizes the difficulty in objectively evaluating the accuracy of a differential diagnosis. Even when the true underlying condition is identified, the quality of the overall differential may be poor due to the omission of important alternative explanations for a patient’s symptoms, or the inclusion of irrelevant diseases. By evaluating differential diagnoses qualitatively using independent judges, we found that considerable disagreement exists in the subjective rating by different individuals, including differential diagnoses of human doctors. This may be due to the fact that a judge’s rating is itself based on personal assessment of the clinical case, which may be prone to error, or due to differences in personal preference for longer or shorter differential diagnoses. Ultimately, there is likely no adequate “gold standard” differential diagnosis, and future work would benefit from assessing the inter-rater agreement between a larger sample of doctors. Further studies using real-world cohorts, for example within a health clinic (Berry et al., 2019), will be required to demonstrate the relative performance of these systems to human doctors in more realistic contexts, where the ability to communicate with a patient is an additional factor in the diagnostic process. Such studies should ideally assess both algorithm performance and user interaction and could follow a multistage process whereby exposure to real-life clinical environments is gradually increased from early observational studies through randomised controlled trials to post-market surveillance during routine operational use (Fraser et al., 2018). Such studies could be informed by existing frameworks for evaluating digital health systems (Stead et al., 1994; Talmon et al., 2009; Jutel and Lupton, 2015; Murray et al., 2016; Millenson et al., 2018).

Finally, we acknowledge the need for standardized evaluation protocols and datasets that allow for a robust and fair comparison of different symptom checkers. No agreed framework currently exists for assessing such AI systems as there is for new drugs or surgical interventions, which presents a challenge for regulating bodies. However, we are encouraged by the recent step taken by the World Health Organization (WHO) and the International Telecommunication Union (ITU) in establishing a Focus Group on Artificial Intelligence for Health (FG-AI4H) with the aim of developing a benchmarking process for applications of AI-assisted healthcare technologies (Wiegand et al., 2019). Through our active participation in this focus group we hope to contribute toward developing an open and transparent framework for evaluating symptom checkers that can build trust in this technology.

Virtual assistants and medical AI technology in general have the potential to reduce costs and improve access to healthcare in resource-poor settings (Guo and Li, 2018). While such technologies may hold the promise of narrowing the gap in healthcare access between high and low income countries, great care must be taken to ensure that algorithms are fair and generalize to different subsets of the population. In particular, lack of diversity in medical datasets has the potential to result in biased algorithms which could widen healthcare inequality (Nordling, 2019). An advantage of Bayesian generative models such as the one used in this study is that it is less susceptible to such biases by incorporating robust epidemiological data for different regions rather than relying solely on datasets which may be biased toward a particular population. A further benefit of using a Bayesian network is that the model is interpretable, since the causal structure of the model allows cause and effect within the system to be observed and understood. This in turn makes the system explainable, which allows triage decisions from the model to be explained to the user in terms of the diseases and symptoms that gave rise to the recommendation. This allows the patient to make a more informed decision about whether to adhere to the advice of the system. This property also enables the model to be internally audited, to understand why particular inferences were made and where improvements to the model need to be targeted.

## Data Availability Statement

The vignettes used for the role play experiment may be found in the **Supplementary Material.**

### ETHICS STATEMENT

Ethical review and approval was not required for the study on human participants in accordance with the local legislation and institutional requirements.

## Author Contributions

AB, YP, KM, and SJ contributed to the conception and design of the study and drafted the manuscript. AB, YP, and SJ contributed to analysis and interpretation of experimental data. KM, JB, DM and DS conducted the experiments and acquired data. All authors contributed to manuscript revision and approved the submitted version.

## Conflict of Interest

AB, YP, KM, JB, DM, DS, MB and SJ are current or former employees of Babylon Health.

The remaining authors declare that the research was conducted in the absence of any commercial or financial relationships that could be construed as a potential conflict of interest.

## Footnotes

1 The term “diagnosis” is used for ease of reference, as shorthand to suggest the matching of symptoms with diseases and/or conditions. However, we are not suggesting that online symptom checkers are diagnosing users, or that the Babylon Chatbot provides a diagnosis.

2 Global Burden of Disease Collaborative Network. Global Burden of Disease Study 2017 (GBD 2017) Results. Seattle, United States: Institute for Health Metrics and Evaluation (IHME), 2018. Available from http://ghdx.healthdata.org/gbd-results-tool

3 The list of conditions modeled by the Triage and Diagnostic System includes the majority of those encountered in General Practice in the United Kingdom (where the study was conducted), but does not include skin conditions, pregnancy-related conditions or pediatric conditions.

4 Dr. Benjamin A. White from Harvard Medical School/Massachusetts General Hospital, Department of Emergency Medicine.
