# Supplementary material for: A Comparison of Artificial Intelligence and Human Doctors for the Purpose of Triage and Diagnosis
Source: Front Artif Intell. 2020 Nov 30;3:543405. doi: 10.3389/frai.2020.543405 (PMC7861270; doi:10.3389/frai.2020.543405)
Supplement: Supplementary file 1 [file datasheet1.pdf]

<u>VIGNETTE 1</u>
Allergic Rhinitis

Demographics:
-Male, Age 35

Presenting Complaint / Initial user input:
-"I've been sneezing and have a stuffy nose."

History, on open questioning:
-Sneezing and stuffy nose occur every spring
-Other symptoms include itchy eyes and runny nose.
-Can be worse after going outdoors.

If asked directly:
-Occasional Cough.
-Itchy sensation in the throat
-Eyes can be red with a little mucus.
-Frequent throat clearing
-No fever, chills or difficulty breathing
-Feels better with Benadryl but it makes him sleepy.
-General malaise with the above symptoms.

FH:
-Father and mother are healthy

PMH:
-Asthma as a child

Allergies: NKDA

VIGNETTE 2
Benign paroxysmal positional vertigo

Demographics:
-Female, Age 60

Presenting Complaint / Initial user input:
-"The room is spinning"

History, on open questioning:
-Sudden onset of room spinning sensation yesterday
-Difficulty with walking due to dizziness
-Worse with moving
-Better lying still
-Vomited once

If asked directly:
-Worse when turning her head to the right.
-Worse getting up out of bed.
-Some improvement since yesterday
-Patient's husband noticed her eyes moving back and forth
-No fever, chills or recent illness.
-No problems with hearing.
-No ear pain or clogged sensation
-No ringing in the ears.
-Similar episode 5 years ago.

FH:
-No relevant FH

PMH:
Diabetes
HLD

Allergies: NKDA

<u>VIGNETTE 4</u>

Lactase Deficiency

Demographics:
Female, age 45

Presenting Complaint / Initial user input:
“My stomach always feels bloated.”

History, on open questioning:
The patient feels bloated most days, often worse after meals.
She has diarrhea fairly frequently.
Sometimes she gets abdominal pain.
Often her stomach makes very loud noises.

Symptoms and risk factors, if asked directly:
She does not have any problems with constipation.
The abdominal pain feels like cramps and is often in the periumbilical area or in the lower quadrants.
Flatulence has been a problem.
She has not had blood in the stool.
Her appetite is normal.
She does not have nausea.
Certain foods such as cheese and milk seem to make symptoms worse.
The bloating does not seem to be related to her menstrual cycle.
She is from Africa.

Duration of symptoms:
These symptoms have been present for a number of years.

Ability to perform ADLS:
The symptoms are rather annoying, but she can perform her ADLs without problems.

Family history:
She remembers her mother complaining of similar symptoms.

Past Medical History:
She is overweight and has borderline high blood pressure.

Drug History:
She does not drink alcohol, smoke, or use drugs.
Allergies:
No known allergies.

VIGNETTE 5
Dislocation of the Shoulder

Demographics
Male, age 35

Presenting Complaint / Initial user input:
“My right shoulder is hurting badly.”

History, on open questioning:
His shoulder started to hurt while playing basketball.
He states that his right shoulder does not look the same as the left one.
He has never had pain in this shoulder prior.

Symptoms and risk factors, if asked directly:
He recalls the pain started after he tried to block a basketball shot.
He has full function of his right hand and forearm, though the right arm seems to want to stay in a funny position.
He tried icing it, but that didn’t help much.
He took some acetaminophen which helped a little.
He does not have any fevers or chills.

Duration of symptoms: The pain started suddenly and has not improved since its onset about 5 hours ago.

Activities of daily living: He has been trying to do all his ADLs with the left hand and arm, because it hurts too much to move his right shoulder.
Family history: There is no relevant family history.
Past Medical History: ACL tear of the right knee requiring surgical repair.
Drug History: He has never smoked. He drinks 4 beers on most weekends. He does not use drugs.
Allergies: Penicillin causes a rash.

<u>VIGNETTE 6</u>
Hordeolum

Demographics:
-Male, Age 18

Presenting Complaint / Initial user input:
-“I have a bump on my eye.”

History, on open questioning:
-The patient reports 1 day of a painful lump on his right eyelid.
-The pain is moderate, only at the lump, and worse with blinking.
-The lump has become more red, warm, and swollen overnight.
-He has had some increased tearing in his eye, “but I’ve been rubbing it a lot.”
-He notes he’s “had a few of these before, but usually they go away on their own.”

If asked directly:
-The lump is at the base of his right upper eyelashes.
-His pain improves with ibuprofen.
-He denies visual changes, blurred vision, flashes or floaters.
-He denies headache or fever.
-He denies any eye pain or injury, or redness in his eye.
-The patient is a student, and has felt well until today, but “my classmates are asking me about it now, so I came in” (i.e. *no problems with performing daily activities).*

Family History:
-No relevant FH

Past Medical History:
-MRSA
-Impetigo

Social History:
-Non-smoker, does not drink alcohol, no recreational drugs.

Allergies: NKDA

<u>VIGNETTE 8</u>
Infective Endocarditis

Demographics:
-Male, Age 28

Presenting Complaint / Initial user input:
-“I’m having a fever and chest pain.”

History, on open questioning:
-The patient reports 2 days of a fever, chills, and chest pain.
-He notes the symptoms started as generalized malaise and fatigue, then he developed shaking chills.
-He has measured a temperature to 39 degrees, and “just can’t stop shaking.”
-His chest pain is moderate in severity, in the center of his chest, and is now associated with shortness of breath.
-He has also developed palpitations.

If asked directly:
-He notes recent intravenous drug use, and licks his needles.
-He has developed aching joints and muscles, and “was sweating all night.”
-He has also noticed a “funny purple rash in my hands.”
-He has never had pain like this before.
-No recent travel, calf pain, or lower extremity swelling.
-Patient is a waiter, and has felt well until 2 days ago, but “I can barely move” (i.e. *severe problems with performing daily activities).*

Family History:
-No relevant FH

Past Medical History:
-Multiple Abscesses
-Septic Thrombophlebitis
-Cellulitis

Social History:
-Non-smoker, no alcohol, daily heroin use.

Allergies: Vancomycin

VIGNETTE 9

Extensor tenosynovitis of thumb (De Quervain tenosynovitis)

Demographics:
-Female, Age 30

Presenting Complaint / Initial user input:
-“My wrist hurts.”

History, on open questioning:
-Gradual onset starting 1 month ago
-Wrist pain located at the base of the thumb
-Pain aggravated by repetitive use of thumb, wrist turning, grasping, pinching, or attempt to make a fist
-Mild swelling at the base of the thumb

If asked directly:
-Had a baby 1 month ago and has been lifting baby repetitively
-Creaking or clicking sound with thumb movement
-Locking (“stop-and-go”) or sticking sensation with thumb movement
-Job requires lots of keyboarding
-Likes to garden and play racket sports

Duration of symptoms:
-1 month

Activities of daily living:
-Some problems performing daily activities

Family history:
-Father has diabetes mellitus, type 2
-Mother has rheumatoid arthritis

Past medical history:
-Normal spontaneous vaginal delivery of newborn
-Overweight
-Trigger finger
-Carpal tunnel syndrome
-No smoking, alcohol, or recreational drugs

Allergies: NKDA

<u>VIGNETTE 10</u>

Benign prostatic hyperplasia

Demographics:
-Male, Age 70

Presenting Complaint / Initial user input:
“Can’t get a good night’s sleep because I have to get up to go to the bathroom”

History, on open questioning:
-Used to wake up once a night to urinate
-Gradually, symptoms have gotten worse over the past 2 years
-Now has to get up 3 or 4 times a night
-“Takes forever” to void

If asked directly:
-Poor urinary flow
-Sometimes very little urine comes out
-Has to wait a long time for urine to come out
-Doesn’t feel like he completely empties his bladder.
-No blood
-No burning on voiding
-Daytime urinary frequency has increased as well.

FH:
-No relevant FH

PMH:
HLD
CAD

Allergies:
NKDA

<u>**VIGNETTE 12**</u>

Acute Myocardial Infarction

Demographics:
-Female, Age 26

Presenting Complaint / Initial user input:
-"I'm having chest pain."

History, on open questioning:
-The patient noticed sudden, sharp, chest pain when bending over to pick up a hairbrush.
-On standing, "I felt like I was going to die and almost passed out."
-After sitting down, she started to feel a bit better, but had a "nagging pressure" in her chest.
-Minimal shortness of breath initially, now resolved.
-Also notes some tingling in her hands, which has also resolved.
-No nausea or vomiting.

If asked directly:
-She has never had an episode like this before.
-No recent travel, calf pain, or lower extremity swelling.
-No recent cough, fever, or recent illness.
-Patient is a student and marathon runner, and exercises daily without chest pain or shortness of breath (i.e. *no problems with performing daily activities).*
-She stayed up late last night studying, and took an Adderall to help her focus.
-This morning, she was rushing to get to her test, but otherwise her morning was typical.

Family History:
-Mother has hypertension; father had some "connective tissue disorder" and died at a young age.
-No other FH

Past Medical History:
-No relevant PMH

Social History:
-Non-smoker, no alcohol, occasional marijuana and Adderall.

Allergies: NKDA

VIGNETTE 13
Trochanteric bursitis

Demographics:
-Female, Age 70

Presenting Complaint / Initial user input:
-"My hip hurts."

History, on open questioning:
-Gradual onset starting 2 weeks ago
-Pain started after slipping at home and falling on the hip
-Moderate to severe pain over the outside point of the hip
-Pain travels to the thigh, knee, and/or buttock on the same side
-Pain is worse when rising from a seated position, feels somewhat better after a few steps, then worsens after walking for half an hour or more

Symptoms and risk factors, if asked directly:
-Pain does not travel to the foot
-Unable to lie on the affected side at night due to pain
-Pain worsens with getting out of a car, walking up stairs, or standing for long periods of time
-Had previous hip surgery and a prosthetic implant in the affected hip

Duration of symptoms:
-2 weeks

Activities of daily living:
-Moderate problems performing daily activities

Family history:
-Father had gout and psoriasis
-Mother had rheumatoid arthritis and osteoporosis

Past medical history:
-Degenerative disc disease of lumbar spine
-Hip osteoarthritis
-Osteoporosis, being treated with alendronate
-Hip fracture status-post open reduction and internal fixation (ORIF)
-No smoking, alcohol, or recreational drugs

Allergies:
-NKDA

<u>VIGNETTE 14</u>
Subdural Hemorrhage

Demographics:
-Female, Age 72

Presenting Complaint / Initial user input:
-“I have a headache.”

History, on open questioning:
-The patient reports 6 hours of a worsening headache.
-It is diffuse, dull, and gradually worsening to “really bad now.”
-She first noticed it when she woke up this morning.
-She also notes a minor fall last night when going to the bathroom, and striking her head on the toilet.
-Since the onset of the headache, she has developed nausea as well.

If asked directly:
-She is feeling more “confused” over the last few hours, and can’t remember her phone number.
-Her vision has also become blurry, “like things are quite lining up right.”
-She has felt dizzy and disoriented as well.
-No chest pain, shortness of breath, recent fever or illness, or diarrhea.
-Patient is a retired nurse, and has been well until today “but I couldn’t really see my lunch” (i.e. *some problems with performing daily activities).*

FH:
-No relevant FH

PMH:
-Hypertension
-Prior stroke, on warfarin
-Recovering alcoholic

SH:
-Non-smoker, no alcohol (sober for 2 years), no recreational drugs.

Allergies: Sulfonamides

<u>VIGNETTE 15</u>
Appendicitis

Demographics:
-Male, Age 23

Presenting Complaint / Initial user input:
-“I’m having abdominal pain.”

History, on open questioning:
-The patient reports 2 days of worsening abdominal pain.
-The pain initially started “all over”, but today has become worse in the right lower abdomen.
-It was dull initially, but now is sharp, and worse with bending over.
-He has had nausea, and one episode of vomiting as well.
-Last night he had shaking chills and night sweats, and has felt “cold all day”.

If asked directly:
-He has lost his appetite as well, and notes some general malaise.
-His pain is much worse if he pushes on the right, lower side of his abdomen.
-He denies diarrhea.
-He denies upper abdominal pain.
-The patient is chef, and has otherwise been well until today, but now “finds the smell of food nauseating” (i.e. *some problems with performing daily activities).*

FH:
-Father has hypertension, mother had cancer.

PMH:
-GERD

SH:
-Non-smoker, drinks 1-2 beers per day, no recreational drugs.

Allergies:
NKDA

VIGNETTE 16
Temporal Arteritis

Demographics:
-Female, Age 72

Presenting Complaint / Initial user input:
-“I’m having a headache and blurred vision.”

History, on open questioning:
-The patient reports 3 hours of headache and blurry vision.
-She first noted the symptoms this morning when she woke up.
-The headache has been moderate, dull, and only on the left side of her head.
-It also hurt in the side of her head when she brushed her hair this morning.
-Then “it started hurting when I was chewing my toast, and I’ve never had that.”
-She then noticed blurry vision, which has worsened since.

If asked directly:
-Today, she also noted generalized malaise and fatigue.
-Just before coming in, she had “chills and a fever I think.”
-She has had diffuse pain in her shoulders and hips over the last 4 days.
-She also had a cough and sore throat last week.
-It hurts most when she rubs her scalp just in front of her left ear.
-No slurred speech, facial droop, tingling or numbness.
-Patient is a retired hair stylist, and has been well up until this morning, but “couldn’t even drive herself to her bridge game” (i.e. *some problems with performing daily activities).*

Family History:
-Father had a stroke, mother had rheumatoid arthritis.

Past Medical History:
-Diabetes
-Hypertension
-Rheumatoid Arthritis
-Peripheral Arterial Disease

Social History:
-Active 1 pack/day smoker, no alcohol, no recreational drugs.

Allergies: NKDA

<u>**VIGNETTE 17**</u>

Medial epicondylitis (golfer's elbow)

Demographics:
-Male, Age 40

Presenting Complaint / Initial user input:
-"My elbow hurts."

History, on open questioning:
-Gradual onset starting 2 months ago
-Pain located on the inner side of elbow or on forearm near the "bony bump" near the elbow
-Stiffness in elbow and weakness in hand or wrist
-Moderate pain triggered by lifting weights, squeezing or pitching a ball, or swinging a golf club or racket
-Mild pain at rest, shaking hands, turning a doorknob, or picking something up with palm facing down

Symptoms and risk factors, if asked directly:
-No obvious injury or trauma to elbow or forearm
-Pain radiates from the inside of the elbow into the forearm or wrist
-Mild numbness or tingling in the ring or little fingers
-Works as a painter, gardener, carpenter, or cook
-Enjoys swimming, baseball, football, and bowling
-Started learning how to play golf 2 months ago

Duration of symptoms:
-2 months

Activities of daily living:
-Some problems performing daily activities

Family history:
-No relevant family history

Past medical history:
-Obese
-Smoker
-Social alcohol use
-No recreational drugs

Allergies:
-NKDA

<u>VIGNETTE 18</u>
Rapid Atrial Fibrillation

Demographics:
-Female, Age 73

Presenting Complaint / Initial user input:
-“I’m having palpitations.”

History, on open questioning:
-The patient reports palpitations for 3 hours.
-They started suddenly after she climbed a flight of stairs this morning.
-She notes that it “feels irregular, and jumping all over the place.”
-She also noticed that her Apple watch has “had numbers from 120 to 150!”
-She has now developed shortness of breath and lightheadedness as well, “I just feel weak all over.”
-No nausea or vomiting.
-Some intermittent palpitations, lightheadedness.

If asked directly:
-She denies chest pain or tightness.
-She has had intermittent “fluttering in my chest” previously, but is has never lasted this long.
-She denies associated nausea, vomiting, or diarrhea.
-No recent travel, calf pain, or lower extremity swelling.
-No recent cough or fever.
-The patient works as a cake decorator, and has felt well otherwise, but now “I get really lightheaded when moving around the kitchen” (i.e. *some problems with performing daily activities).*

Family History:
-Mother had heart disease, father had prostate cancer.

Past Medical History:
-Diabetes
-Hypertension
-COPD

Social History:
-Active 1 pack/day smoker, drinks 2-3 glasses of wine per day, no recreational drugs.

Allergies: Soy

VIGNETTE 20
Iron Deficiency Anemia

Demographics:
-Female, Age 25

Presenting Complaint / Initial user input:
“Tired”

History, on open questioning:
-More tired over the past 6 months
-Sleeps well but still tired
-Some shortness of breath running
-Can’t run as far as she used to

If asked directly:
-Heavy menstrual periods
-Became a vegetarian to lose weight
-No chest pain.
-Does not take iron supplements
-Eats spinach but is not sure how much iron she gets in her diet

FH:
-No relevant FH

PMH:
Menorrhagia
Obesity

Allergies: NKDA

<u>VIGNETTE 21</u>

Cyst of Bartholin's Gland Duct

Demographics:
Female, age 58

Presenting Complaint / Initial user input:
“There is a lump near the opening of my vagina.”

History, on open questioning:
There is a soft lump to the left of the opening to the vagina.
It started out smaller and didn’t really bother her, but in the last 2 weeks, it has grown.
Now it is uncomfortable to sit or walk.

Symptoms and risk factors, if asked directly:
There is no discharge from the lump.
There is no discharge from the vagina.
It is uncomfortable to have intercourse.
She denies any fevers or chills.
She recalls having a similar problem a few years ago, but cannot recall which side.
She denies having had any trauma to the area.
She has not needed to take any pain medications for the discomfort.
She does not have any pelvic pain.

Duration of symptoms:
She has first noticed the swelling about 5 weeks ago.

Activities of daily living:
She has no problems performing her ADLs.

Family history:
No relevant family history

Past Medical History:
obesity, diabetes mellitus type 2

Drug History:
She has mixed drinks and cocktails when she goes out with friends. She smokes ½ a pack of cigarettes a day and has been doing so for 25 years. She denies drug use.

Allergies:
Amoxicillin gives her a rash.

<u>**VIGNETTE 22**</u>

Acute lower urinary tract infection

Demographics:
- Female, age 22

Presenting Complaint / Initial user input:
"I'm having burning with urination"

History, on open questioning:
- Symptoms started about 2 days ago
- Along with dysuria she also has increased urinary frequency and urinary urgency
- Mild dysuria initially but now with more significant dysuria
- Urinary urgency now occurring every 1 hour or so but only able to void small amounts of urine each time

Symptoms and risk factors, if asked directly:
- Has also started to have intermittent crampy 5/10 suprapubic pain over the last few hours
- New gross hematuria over the last few hours
- No fevers/chills
- No flank pain
- No vaginal pruritis or vaginal discharge
- No constipation or diarrhea or rectal bleeding
- Last menstrual period was 1 week ago
- No history of prior STDs

Duration of symptoms:
Days

Activities of daily living:
Some problems performing daily activities

Family history:
No relevant family history

Past Medical History:
None

Past Social History:
non-smoker, does not drink alcohol, no other drug use history, married and in a monogamous relationship

Drug History:
None
Allergies:
NKDA

<u>VIGNETTE 24</u>
Synovial cyst of popliteal space (Baker's cyst)

Demographics:
-Male, Age 40

Presenting Complaint / Initial user input:
-"There is a bulge behind my knee."

History, on open questioning:
-Gradual onset starting 1 month ago
-Swelling, fullness, and a feeling of tightness behind the knee
-Mild pain and stiffness in the knee
-Pain is worse when physically active and if the knee is fully flexed or extended

Symptoms and risk factors, if asked directly:
-No redness or warmth over the swollen area
-No pain or swelling of the calf on the affected side
-No difficulty breathing or rapid heart rate
-Likes to play football, tennis, and basketball
-Tore his meniscus (in the affected knee) playing basketball about 1 month ago

Duration of symptoms:
-1 month

Activities of daily living:
-Some problems performing daily activities

Family history:
-Father has gout
-Mother had a deep vein thrombosis (DVT)

Past medical history:
-Torn meniscus
-Anterior cruciate ligament (ACL) injury
-Knee osteoarthritis
-No smoking, alcohol, or recreational drugs

Allergies:
-NKDA

<u>**VIGNETTE 25**</u>
Gout of hallux

Demographics:
- Male, Age 53

Presenting Complaint / Initial user input:
“I’m having new onset of pain in my left foot”

History, on open questioning:
- Pain started overnight and has gotten worse today
- Pain located in left foot big toe
- Big toe is swollen, red, and warm to touch
- Severe (8/10) and sharp pain
- Pain is making it difficult to walk
- No history of similar episodes in the past

Symptoms and risk factors, if asked directly:
- No recent trauma of the L foot
- No other joint involved
- No fevers/chills
- No new rashes
- No vision changes
- No dysuria or urethral discharge
- Increased weight over the last year (10 pounds)
- No recent tick bite
- History of HTN, recently started on hydrochlorothiazide a few weeks ago
- No history of prior L foot surgery or hardware
- No history of IV drug use
- No history of immunosuppression and not on any immunosuppressant medications
- Drinks 10-14 beers per week
- Married for 30 years, no new sexual partners

Duration of symptoms:
hours-days

Activities of daily living:
Some problems performing daily activities

Family history: no relevant FH

Past Medical History:
HTN

Past Social History:
non-smoker, drinks 10-14 beers/week, no other drug use history
Drug History:
Hydrochlorothiazide

Allergies:
NKDA

<u>VIGNETTE 26</u>

Guillain-Barre Syndrome

Demographics:
-Female, Age 62

Presenting Complaint / Initial user input:
-“I’m feeling weak.”

History, on open questioning:
-The patient reports 3 days of worsening weakness in her extremities.
-“It’s like my legs have become really heavy for some reason.”
-She first noticed it as “tingling when climbing stairs”, but “now I can’t even stand without support.”
-Some associated generalized malaise, “It’s like the weakness is climbing up my body.”
-Denies trauma to her back, or low back pain.

If asked directly:
-Today, she also noted weakness in her arms when picking up her cup of coffee.
-She’s also developed shortness of breath, that doesn’t resolve with sitting down.
-“Even my head feels heavy.”
-She does note a recent sore throat and fever, which resolved last week.
-No loss of bowel or bladder control.
-No chest pain, palpitations, or lightheadedness.
-Patient is a retired chemist, and has been well up until 2 days ago, but “now I feel like I’m crippled” (i.e. *severe problems with performing daily activities).*

FH:
-Father had coronary artery disease, mother had a stroke.

PMH:
-Diabetes
-Hypertension
-Rheumatoid Arthritis

SH:
-Active 1 pack/day smoker, no alcohol, no recreational drugs.

Allergies: NKDA

<u>**VIGNETTE 28**</u>

Subarachnoid Hemorrhage

Demographics:
-Male, Age 42

Presenting Complaint / Initial user input:
-“I have a really bad headache.”

History, on open questioning:
-The patient has a severe headache; started while chopping wood.
-The pain came on suddenly, and is “pounding” when he bends over.
-Initially he noted some blurred vision, but thought it was because his eyes were tearing.
-He also developed nausea and one episode of vomiting.

If asked directly:
-He notes that the he was feeling fine today, but then “it was like someone hit me in the head with a bat.”
-He took 4 tablets of ibuprofen without relief.
-He also notes mild lightheadedness.
-He thinks he may have some tingling in his arms.
-This is the “worst headache I’ve ever had.”
-No recent fevers, chills, or trauma to the head.
-Patient is a construction worker and labors daily without headaches.
-However today, he describes the headache as “debilitating” (i.e. *unable to perform daily activities).*

FH:
-Mother and father have hypertension.

PMH:
-Hypertension
-Hyperlipidemia
-Peripheral Vascular Disease

SH:
- Non-smoker, drinks 1-3 beers per day. No recreational drugs.

Allergies: PCN

<u>VIGNETTE 29</u>

Acute Exacerbation of Chronic Obstructive Airways Disease

Demographics:
-Male, Age 76

Presenting Complaint / Initial user input:
-“I’m having shortness of breath.”

History, on open questioning:
-The patient reports 1 day of worsening shortness of breath.
-He first noticed a worsening cough yesterday, then he woke up this morning with “lots of wheezing.”
-He notes he “feels like I’m breathing through a straw.”
-Over the past few hours, he has had to sit up and lean forward, and purse his lips “to get any air at all.”
-He also notes some lightheadedness, “I felt like I was going to pass out.”

If asked directly:
-He reports intermittent symptoms like this for months, “but it’s a lot worse in the winter.”
-He endorses an increasingly productive cough over the last few days.
-He has taken his inhalers, and that helped some, but only briefly.
-He denies chest pain, recent travel, calf pain, or lower extremity swelling.
-He denies fevers, chills, nausea, or vomiting.
-The patient is college professor, and felt well until yesterday, but notes “I’m too winded to even speak, let alone teach” (i.e. *severe problems with performing daily activities).*

Family History:
-Father has coronary artery disease, mother has COPD.

Past Medical History:
-COPD
-Hypertension

Social History:
-Active 1 pack/day smoker, no alcohol, no recreational drugs.

Allergies: PCN

<u>**VIGNETTE 30**</u>

Atrial Fibrillation

Demographics:
-Female, Age 65

Presenting Complaint / Initial user input:
“Palpitations”

History, on open questioning:
-Palpitations for 10 minutes to 1 hour at times
-“My hear feels like it is beating out of my chest”
-Started 1 year ago
-More frequent
-Now occurs every few days
-Tired during episodes
-Worse with drinking alcohol and coffee so she stopped drinking these

If asked directly:
-No chest pain or shortness of breath
-No fainting
-No drug use.
-No history of strokes or mini-strokes (TIAs)

FH:
Heart disease

PMH:
HTN
Diabetes

Allergies: NKDA

VIGNETTE 31
Kidney stone

Demographics:
- Male, age 29

Presenting Complaint / Initial user input:
"I'm having severe back pain"

History, on open questioning:
- Pain located left lower back
- Pain started about 6 hours ago
- Intermittent waves of severe sharp pain lasting about 30 minutes
- Pain level gets up to 9/10 with intensity

Symptoms and risk factors, if asked directly:
- Increased urinary frequency, urgency, dysuria and gross hematuria
- No association of pain with eating
- No association of pain with positional changes
- Mild relief of pain with naproxen
- No nausea or vomiting
- No fevers/chills
- No constipation or diarrhea or rectal bleeding
- No cough, shortness of breath, chest pain
- No rashes
- No recent trauma or injuries, no recent change in level of physical activity
- No urethral discharge, no history of prior STDs

Duration of symptoms:
Hours

Activities of daily living:
Severe problems performing daily activities

Family history:
FH kidney stones in dad

Past Medical History:
None

Past Social History:
non-smoker, does not drink alcohol, no other drug use history, married and in a monogamous relationship

Drug History:
Vitamin C supplementation

Allergies:
NKDA

VIGNETTE 32

Acute Exacerbation of Chronic Congestive Heart Failure

Demographics:
-Female, Age 75

Presenting Complaint / Initial user input:
-"I'm having shortness of breath."

History, on open questioning:
-The shortness of breath has been intermittent for months, worse over the last 3 days.
-She notes that she "can't even go to the bathroom without feeling short of breath."
-Today, "it got bad even sitting on the couch when I tried to put my legs up."
-Patient notes lightheadedness as well, and some palpitations.
-Also reports increased swelling in both legs, "soft and squishy."
-Denies chest pain.

If asked directly:
-She has been sleeping with more pillows over the last week, and now sleeps in her armchair (orthopnea).
-Last night, she got up suddenly ("I felt like I was suffocating"), and ran to the window for air (PND).
-Swelling in her legs is becoming painful as on both sides.
-"I'm peeing a lot at night too."
-No recent travel, or focal calf pain.
-No recent cough, fever, nausea, vomiting, or diarrhea.
-Patient is a school teacher, and has had intermittent symptoms for months (i.e. *some problems with performing daily activities).*

Family History:
-Mother had a history of "heart disease," father died of stroke.

Past Medical History:
-Hypertension
-Coronary Artery Disease
-Prior Myocardial Infarction

Social History:
-Non-smoker, drinks 3-4 beers per day, no recreational drugs.

Allergies: PCN

VIGNETTE 33
Pyelonephritis

Demographics:
- Female, age 30

Presenting Complaint / Initial user input:
"I'm having nausea and back pain"

History, on open questioning:
- Pain located deep in the right side of her lower back
- Pain started about 24 hours ago
- Dull and aching pain that is up to 5/10 in intensity

Symptoms and risk factors, if asked directly:
- 2-3 days ago started to have increased urinary frequency and burning with urination
- 1 day of nausea, vomiting, decreased appetite
- 1 day of generalized fatigue and malaise
- No association of pain with eating
- Subjective fevers and chills over the last few hours
- No constipation or diarrhea or rectal bleeding
- No gross hematuria
- No cough, shortness of breath, chest pain
- No rashes
- No recent trauma or injuries, no recent change in level of physical activity
- Last menstrual period was 1 week ago
- No vaginal discharge, no history of prior STDs

Duration of symptoms: Days

Activities of daily living:
Some problems performing daily activities

Family history:
No relevant family history

Past Medical History:
History of recurrent UTIs

Past Social History: non-smoker, does not drink alcohol, no other drug use history, married and in a monogamous relationship

Drug History:
Treated with nitrofurantoin about 2 weeks ago for a UTI

Allergies:
NKDA

VIGNETTE 34
Acute Pancreatitis

Demographics:
- Female, Age 45

Presenting Complaint / Initial user input:
"I'm having new abdominal pain"

History, on open questioning:
- Pain started a few hours ago
- Pain located in the mid-upper abdomen with radiation to the back
- Severe pain with 9/10 intensity
- Pain improved with sitting up and bending forward, worse with laying down

Symptoms and risk factors, if asked directly:
- Severe nausea and vomiting
- Unable to tolerate food or liquids due to vomiting
- Unable to eat or drink anything this morning due to nausea
- No fevers/chills
- No constipation or diarrhea
- No cough, shortness of breath, chest pain
- No dysuria, hematuria, flank pain
- No rectal bleeding or hematemesis
- No jaundice
- No prior abdominal surgery
- No recent travel or exotic food ingestion
- No recent NSAID or aspirin use

Duration of symptoms:
Hours

Activities of daily living:
Severe problems performing daily activities

Family history:
No relevant family history

Past Medical History:
None

Past Social History:
non-smoker, drinks about 7 glasses of alcohol per week, no other drug use history

Drug History:
None

Allergies: NKDA

<u>**VIGNETTE 35**</u>

Lateral epicondylitis (tennis elbow)

Demographics:
-Male, Age 40

Presenting Complaint / Initial user input:
-“My elbow hurts.”

History, on open questioning:
-Gradual onset starting 2 months ago
-Pain located on the elbow or on forearm near the “bony bump” outside the elbow
-Moderate pain triggered by lifting, turning a screwdriver, or hitting a backhand in tennis
-Mild pain at rest, shaking hands, holding a cup of coffee, using a key, or turning a doorknob

Symptoms and risk factors, if asked directly:
-No obvious injury or trauma to elbow or forearm
-Pain radiates from the outside of the elbow into the forearm or wrist
-Works as a plumber, painter, carpenter, butcher, or cook
-Started learning how to play tennis 2 months ago

Duration of symptoms:
-2 months

Activities of daily living:
-Some problems performing daily activities

Family history:
-No relevant family history

Past medical history:
-No relevant past medical history
-No smoking, alcohol, or recreational drugs

Allergies:
-NKDA

<u>VIGNETTE 36</u>
Ovarian Torsion

Demographics:
-Female, Age 22

Presenting Complaint / Initial user input:
-"I'm having abdominal pain."

History, on open questioning:
-The patient reports 1 hour of lower abdominal pain.
-The pain started suddenly just following sexual intercourse this evening.
-She notes "it came out of nowhere" and is sharp and severe "like someone stabbed me."
-Since then, she has developed nausea and 3 episodes of vomiting.
-She denies vaginal bleeding or discharge.

If asked directly:
-The pain is "the worst I've ever had."
-Her pain is worse when she pushes "very low down" on her left abdomen.
-She has had intermittent "crampy pain in that area before."
-She also notes a feeling of general malaise.
-She has also developed discomfort with urinating.
-She denies recent fever, chills, or flank pain.
-The patient is medical student and has felt well until today, but now "the pain is so bad I can't move" (i.e. *severe problems with performing daily activities).*

FH:
-Mother has ovarian cysts, father had testicular cancer.

PMH:
-Polycystic Ovarian Syndrome
-Undergoing hormone therapy for infertility
-S/p appendectomy

SH:
-Non-smoker, no alcohol, occasional Adderall.

Allergies: NKDA

<u>VIGNETTE 37</u>
Retinal Artery Occlusion

Demographics:
-Male, Age 77

Presenting Complaint / Initial user input:
-"I can't see."

History, on open questioning:
-The patient notes sudden loss of vision in his right eye 45 minutes ago.
-He was "just sitting there at my workbench, and someone pulled a shade down over my eye."
-He had blurry vision and a few sensations of "flashy lights" just before he lost vision.
-"Now it's totally black, I can't see anything."
-He denies headache.

If asked directly:
-He notes that the he was feeling fine today before these symptoms.
-He denies any eye pain or injury.
-His left eye vision is intact.
-He denies slurred speech, facial droop, tingling, numbness, or weakness.
-No recent fevers, chills, or trauma to the head.
-Patient is a retired astronaut, and has felt well until today, but "then I couldn't see the fly I was tying" (i.e. *some problems with performing daily activities).*

Family History:
-Father had a stroke, mother had heart disease and cancer.

Past Medical History:
-Diabetes (type II)
-Hypertension
-Hyperlipidemia
-Coronary artery disease
-Peripheral vascular disease

Social History:
- Active 2 pack/day smoker, does not drink alcohol, no recreational drugs.

Allergies: NKDA

<u>VIGNETTE 38</u>
Acute Retention of Urine

Demographics:
-Male, Age 78

Presenting Complaint / Initial user input:
-"I can't pee."

History, on open questioning:
-The patient reports 12 hours of an inability to urinate.
-He notes a "dribbling stream last night, but now nothing is coming out."
-This morning, he started to have midline lower abdominal discomfort.
-It was mild and crampy at first, but now is more severe.
-He also notes abdominal distension, "it's like I'm pregnant!"

If asked directly:
-He notes increased discomfort with pushing on his lower abdomen.
-His abdominal distension is entirely in the lower abdomen.
-He denies blood in his urine, flank pain, or fever.
-He denies nausea, vomiting, or diarrhea.
-He recently started taking more of his anxiety and allergy medications as well.
-The patient is commercial fisherman, and has felt well until today, "but this pain is making it hard to walk, let alone fish." (i.e. *some problems with performing daily activities).*

Family History:
-Father had BPH and prostate cancer, mother had diabetes.

Past Medical History:
-Benign Prostatic Hyperplasia
-Diabetes
-Depression and anxiety
-Atopic Dermatitis

Social History:
-Active 1 pack/day smoker, drinks 1-2 beers per day, occasional marijuana.

Allergies: Sulfonamides

VIGNETTE 39
Normal Pressure Hydrocephalus

Demographics:
Male, age 82

Presenting Complaint / Initial user input:
His spouse says, “He has been urinating on himself.”

History, on open questioning:
The patient has been incontinent of urine for the past 2 weeks.
He walks very slowly now and shuffles. His spouse wonders if he is incontinent because he cannot make it to the bathroom on time.

Symptoms and risk factors, if asked directly:
He denies dysuria.
He had urinary urgency prior, but now he is incontinent.
He denies fevers or chills.
He does not have any back pain.
His spouse says he is very forgetful. He doesn’t seem to care about anything. His wife wonders if he is depressed.
He occasionally complains of headaches.
His vision is unchanged.
He takes more steps than the average person to turn and has fallen while trying to turn.
He does not have a tremor.
He was a math professor.

Duration of symptoms:
The gait change started about 2 years ago. The urinary urgency started about the same time. His cognitive decline started about a year ago.

Activities of daily living:
He has trouble performing many of the ADLs without assistance. He has not been able to help with the finances for a year now.

Family history:
No family history of Parkinson’s or dementia.

Past Medical History:
hypertension, coronary artery disease, peripheral arterial disease.

Drug History:
He never smoked. He drinks a glass of wine about 3 nights a week. He never used drugs.

Allergies:
No known allergies.

VIGNETTE 40
Acute exacerbation of chronic congestive heart failure

Demographics:
-Male, Age 60

Presenting Complaint / Initial user input:
"Can't breath"

History, on open questioning:
-Legs are swollen
-Wakes up at night short of breath
-Can't lie flat or he feels like he can't breath

If asked directly:
-Gained 10 lbs over the past week
-Had bacon and other salty food for brunch for Father's Day.
-Wasn't able to pick up his medications due to lack of money for the co-pay
-Feels wheezy
-Shoes don't fit

FH:
-Heart disease
-COPD

PMH:
HLD
CAD
Diabetes Type 2
Smoking

Allergies: NKDA

<u>VIGNETTE 41</u>
Common Bile Duct Calculus

Demographics:
Female, age 45

Presenting Complaint / Initial user input:
"I have pain in the mid-abdominal area."

History, on open questioning:
The pain comes in waves.
The pain is worse after eating.
Nausea is present when the pain is at its worst.

Symptoms and risk factors, if asked directly:
The episodes of pain can last all day.
The pain can be so bad that she gets the sweats.
The pain sometimes radiates to the shoulder blade area.
The pain seems to come on after eating heavy, fatty meals.
The pain does not seem to change with movement.
Having a bowel movement does not seem to affect the pain.
She has noticed some yellowing of her eyes.
She denies fevers or chills.
The urine is dark and the stool pale.

Duration of symptoms:
She has been having these episodes periodically for months. They have gotten more frequent in the past 2 weeks.

Activities of daily living:
She is able to perform her ADLs, but cannot focus on her work when the episodes hit her hard.

Family history: No relevant family history.

Past Medical History:
obesity with BMI of 41

Drug History:
2 pack-year smoking history, quit 5 years ago, 1 alcoholic beverage about 3 nights a week. No history of drug use.

Allergies:
NKDA

VIGNETTE 42
Ruptured Abdominal Aortic Aneurysm

Demographics:
-Male, Age 53

Presenting Complaint / Initial user input:
-“I think I’m dying.”

History, on open questioning:
-The patient reports severe abdominal pain that started 15 minutes ago.
-He was “doing a few sit-ups, and then it was like someone stabbed me.”
-His pain is sharp, located in the mid-abdomen, and is “the worst I’ve ever had by far.”
-It was so bad he passed out, and his personal trainer called 911.
-He has since developed lightheadedness, nausea, and feels sweaty.
-His trainer said he “looked really pale and ashen.”

If asked directly:
-He notes that this pain “has a tearing quality to it.”
-The pain radiates to his back.
-He is now noticing tingling in his legs.
-He denies chest pain or shortness of breath.
-He denies recent fevers, chills, or trauma to the head.
-The patient is professional wrestler, and felt well until 15 minutes ago, but “in all my years I’ve never hurt this bad” (i.e. *unable to perform daily activities).*

Family History:
-Mother has diabetes and mixed connective tissue disorder, father died of a ruptured aneurysm.

Past Medical History:
-Hypertension
-Hyperlipidemia
-Peripheral Vascular Disease

Social History:
-Active 1 pack/day smoker, drinks 1-3 beers per day. Occasional cocaine.

Allergies: PCN

<u>**VIGNETTE 43**</u>
Costochondritis

Demographics:
-Male, Age 33

Presenting Complaint / Initial user input:
-“I’m having chest pain.”

History, on open questioning:
-The patient has had sharp, constant, central chest pain over the last 2 days.
-The pain is exquisitely worse with pushing on the center of his chest, and sometimes with a deep breath.
-Moderate in severity.
-It does not radiate anywhere, and is not associated with shortness of breath.
-No nausea or vomiting.
-Other than being distracted, he is able to perform his normal ADLs.

If asked directly:
-He notes a cough and sore throat over the last week, which is getting better over the last 3 days.
-It was partially relieved by ibuprofen yesterday evening, and it helped him sleep, but returned this morning.
-He has never had pain like this before.
-No recent travel, calf pain, or lower extremity swelling.
-No fevers, chills.
-No malaise, palpitations, or lightheadedness.
-Patient is a student and exercises daily without chest pain or shortness of breath (i.e. *no problems with performing daily activities).*

Family History:
-No relevant FH

Past Medical History:
-No relevant PMH

Social History:
-Non-smoker, no alcohol, occasional marijuana.

Allergies: NKDA

<u>**VIGNETTE 44**</u>

Pyelonephritis

Demographics:
-Female, Age 27

Presenting Complaint / Initial user input:
-"I'm having pain when I pee."

History, on open questioning:
-The patient reports 4 days of pain with urinating.
-She notes a "burning sensation when I urinate, and I'm going a lot more often."
-She has also developed a crampy sensation in the middle of her lower abdomen.
-Over the last day, she has developed "pain in the sides of my back."
-"I think I have a fever too, and I've been shaking with chills."
-Her urine has been "cloudy and foul smelling" for the last 3 days.

If asked directly:
-She notes generalized malaise and nausea, and one episode of vomiting.
-Her flank pain is dull, and worse with pushing just below her ribs.
-Her pain is also worse when she pushes "very low down" in the middle of her abdomen.
-She has a strong, persistent urge to urinate, but "very little comes out."
-The patient is pharmacist and has felt well until the last few days, but she called in sick yesterday "because I just feel washed out" (i.e. *some problems with performing daily activities).*

FH:
-Mother had ovarian cancer.

PMH:
-Frequent UTIs
-Diabetes
-HIV

SH:
-Active 1 pack/day smoker, occasional alcohol, no recreational drugs.

Allergies: Fluoroquinolones

VIGNETTE 45
Ischemic Stroke

Demographics:
-Male, Age 84

Presenting Complaint / Initial user input:
-“My face is drooping.”

History, on open questioning:
-The patient notes that “right side of my face in drooping”; started 1 hour ago.
-The patient was shaving, and noticed numbness in his face this morning.
-Then he noted his coffee spilled out of the side of his mouth.
-His coffee cup also felt heavier than normal (RUE weakness).
-He denies headache.

If asked directly:
-He notes that the he was feeling fine today before these symptoms.
-He also had trouble describing symptoms to his wife, because “I couldn’t find the words” (word finding difficulty).
-His wife notes his words are now slurring “like he’s drunk,” and he doesn’t understand her very well.
-He also stumbled while getting into the car and said his right leg “wasn’t working right.”
-No neck pain or chest pain.
-No recent fevers, chills, or trauma to the head.
-Patient is a retired physicist, and goes for long walks multiple times per week.
-However over the last hour his symptoms are progressing, and he seems confused (i.e. *severe problems with performing daily activities).*

FH:
-Father had a stroke, mother had heart disease and cancer.

PMH:
- Diabetes (type II)
- Hypertension
- Hyperlipidemia
- Coronary artery disease

SH:
- Active 1 pack/day smoker, does not drink alcohol, no recreational drugs.

Allergies: Aspirin

<u>VIGNETTE 46</u>
Upper Gastrointestinal Bleed

Demographics:
-Male, Age 45

Presenting Complaint / Initial user input:
-"I'm vomiting blood."

History, on open questioning:
-The patient reports 1 hour of vomiting blood.
-The symptoms started suddenly after eating dinner.
-He noted nausea, then a sudden need to vomit.
-He has vomited "at least 10 times," and each has had "a lot of blood."
-He has now developed sharp upper abdominal pain, and lightheadedness.
-"I feel like my heart is racing too."

If asked directly:
-This has happened a few times previously, but "only small amounts."
-It has been accompanied by shortness of breath and generalized malaise.
-He denies diarrhea, or lower abdominal pain.
-He denies recent fever or chills.
-The patient is recovery coach at an alcohol detox center, and has otherwise been well until 1 hour ago, but now "can't do anything but vomit" (i.e. *unable to perform daily activities).*

Family History:
-Father is a recovering alcoholic, mother had gastric cancer.

Past Medical History:
-Diabetes
-Hypertension
-Coronary Artery Disease
-Cirrhosis

Social History:
-Active 1 pack per day smoker, drinks "a pint" of whisky per day, no recreational drugs.

Allergies: NKDA

VIGNETTE 47
Olecranon bursitis

Demographics:
-Male, Age 40

Presenting Complaint / Initial user input:
-“My elbow is swollen.”

History, on open questioning:
-Gradual onset starting 1 week ago
-Pain and swelling started after slipping on a concrete basketball court and falling hard on the elbow
-Pain is mild to moderate, but swelling is biggest concern
-Unable to put on long-sleeved shirts because elbow is too swollen
-Elbow range of motion is limited due to swelling

Symptoms and risk factors, if asked directly:
-No fever
-No redness or warmth over the elbow
-Likes to play street basketball
-Works as a plumber or heating and air conditioning technician (has to crawl in tight spaces and lean on elbow for long periods of time)

Duration of symptoms:
-1 week

Activities of daily living:
-Some problems performing daily activities

Family history:
-Father is a smoker and has COPD
-Mother has rheumatoid arthritis

Past medical history:
-Asthma
-Gout
-Social smoker and alcohol user
-No recreational drugs

Allergies:
-NKDA

VIGNETTE 48
Plantar fasciitis

Demographics:
-Female, Age 40

Presenting Complaint / Initial user input:
-“My heel hurts.”

History, on open questioning:
-Gradual onset starting 1 month ago
-“Stabbing” pain in the bottom of the foot near the heel
-Prolonged standing or walking increases the pain
-Sitting relieves the pain, but pain returns upon rising

Symptoms and risk factors, if asked directly:
-No obvious trauma, fall, or twisting injury to foot
-Pain most intense when rising from resting position, especially with first steps out of bed in the morning
-Pain is worse after exercise, not during it
-Works as a teacher and stands for long periods
-Enjoys long-distance running, ballet dancing, and aerobic dance
-Bought new running shoes 1 month ago

Duration of symptoms:
-1 month

Activities of daily living:
-Some problems performing daily activities

Family history:
-Father has gout
-Mother has diabetes mellitus, type 2

Past medical history:
-Obese
-Diabetes mellitus, type 2
-Hypertension
-Flat foot (pes planus)
-No smoking, alcohol, or recreational drugs

Allergies: NKDA

VIGNETTE 49
Cauda Equina Syndrome

Demographics:
-Male, Age 62

Presenting Complaint / Initial user input:
-“I’m having back pain.”

History, on open questioning:
-The patient reports 2 weeks of worsening back pain.
-The pain initially started as a “dull, nagging pain in my lower back.”
-It has now become severe over the last day, and is shooting down his legs.
-He also notes numbness in both legs, and trouble walking.
-He denies trauma to his back.

If asked directly:
-His difficulty with walking is because “both my legs feel weak.”
-Today he has developed numbness around his genitals.
-He also notes difficulty holding his urine, and is wearing a diaper.
-He denies abdominal pain.
-The patient is construction worker, and has had mild back pain before, but “now I can’t even stand up” (i.e. *severe problems with performing daily activities).*

FH:
-Father had spinal stenosis, mother had cancer.

PMH:
-Lumbar disc disease
-Spinal stenosis

SH:
-Non-smoker, drinks 1-2 beers per day, no recreational drugs.

Allergies: NKDA

<u>VIGNETTE 50</u>

Obstructive Sleep Apnea Syndrome

Demographics:
-Male, Age 45

Presenting Complaint / Initial user input:
“Sleepy all the time”

History, on open questioning:
-Can fall asleep any time
-Fell asleep at the stoplight
-Doesn’t feel well rested in the morning after sleeping 8 hours.
-He is embarrassed that he falls asleep during meetings
-Worse over the past year

If asked directly:
-His wife complains that he snores
-Gained 20 lbs over the past year
-Had to buy new shirts as his neck size increased
-No problems falling asleep or staying asleep.
-Keeps a regular sleep schedule but naps during the day
-His wife reports he sounds like he is choking when he is sleeping and stops breathing for a few seconds.

FH:
Obesity

PMH:
HTN
Diabetes
Obesity

Allergies: NKDA

VIGNETTE 51
Acute Myocardial Infarction

Demographics:
-Female, Age 85

Presenting Complaint / Initial user input:
-"I'm having shortness of breath."

History, on open questioning:
-The shortness of breath has been intermittent for months, but just got a lot worse while climbing stairs today.
-Denies chest pain, but is having a moderate pressure-like discomfort in her epigastrium.
-Some associated generalized malaise, "I just feel weak all over."
-No nausea or vomiting.
-Some intermittent palpitations, lightheadedness.

If asked directly:
-Today, she "got really breathless and lightheaded" as she was climbing stairs (near syncope).
-Shortness of breath didn't resolve with sitting down, and she started to feel sweaty.
-No recent travel, calf pain, or lower extremity swelling.
-No recent cough, fever, or diarrhea.
-Patient is retired, and has had intermittent symptoms for months, but she "just thought it was my GERD" (i.e. *some problems with performing daily activities)*
-Also noting difficulty carrying groceries into her apartment.

Family History:
-Mother had cancer.
-No other FH: "this was back before we kept track of those things."

Past Medical History:
-Diabetes
-Hypertension
-GERD

Social History:
-Active 1 pack/day smoker, no alcohol, no recreational drugs.

Allergies: NKDA

<u>**VIGNETTE 52**</u>
Acute Cholecystitis

Demographics:
-Female, Age 45

Presenting Complaint / Initial user input:
-"I'm having abdominal pain."

History, on open questioning:
-The patient reports 2 hours of significant right upper abdominal pain.
-She has intermittently has pain for months, but today "it got really bad."
-It started just after having fish and chips for lunch with some friends.
-The pain is sharp, and much worse with pushing on the right side.
-She has had nausea and multiple episodes of vomiting.

If asked directly:
-She also notes "a weird pain in the back of my right shoulder."
-This is the worst abdominal pain she's ever had.
-It has been accompanied by sweating and some shaking chills.
-Her intermittent pains over the last few months have been brief and resolved on their own.
-She denies diarrhea, or lower abdominal pain.
-The patient is nurse at a fertility clinic, and has otherwise been well until the last 2 hours, but now "can't even walk it's so bad" (i.e. *severe problems with performing daily activities).*

FH:
-Father and mother have morbid obesity and diabetes.

PMH:
-Morbid Obesity
-Diabetes
-Hyperlipidemia

SH:
-Active 1 pack per day smoker, drinks occasionally, no recreational drugs.

Allergies: PCN

<u>**VIGNETTE 53**</u>

Acute Pancreatitis

Demographics:
-Female, Age 31

Presenting Complaint / Initial user input:
-“I’m having abdominal pain.”

History, on open questioning:
-The patient reports 3 days of worsening upper abdominal pain.
-The pain is “right in the middle”, and is nagging and constant.
-Sometimes the pain “goes to my back too”.
-The pain is sharp and severe, and worse with pressing on her upper abdomen.
-She has had nausea and multiple episodes of vomiting.

If asked directly:
-The pain got much worse after she tried to eat eggs and bacon this morning.
-It did get a little better with bending over while vomiting.
-Over the last few hours she’s also noting subjective fever and chills.
-She also notes some general malaise.
-She denies diarrhea, right sided or lower abdominal pain.
-The patient is bartender, and has otherwise been well until the last 3 days, but now “can’t even get off the couch” (i.e. *severe problems with performing daily activities).*

FH:
-Father has diabetes; mother has gallstones (cholelithiasis).

PMH:
-Cholelithiasis
-Hyperlipidemia

SH:
-Non-smoker, drinks 2-3 vodka & Red Bulls per day, no recreational drugs.

Allergies: Sulfonamides

VIGNETTE 54
Acute Exacerbation of Asthma

Demographics:
-Male, Age 16

Presenting Complaint / Initial user input:
-"I'm having shortness of breath."

History, on open questioning:
-The patient reports 4 hours of worsening shortness of breath.
-It started suddenly after playing ice hockey outside today.
-He first noticed some mild shortness of breath, but now it "feels like I'm breathing through a straw."
-He endorses significant wheezing, "it sounds funny when I breathe."
-He also notes some lightheadedness, "I felt like I was going to pass out."

If asked directly:
-He reports a "tight" sensation in his chest, but no real chest pain or palpitations.
-He endorses a non-productive cough over the last few days.
-"I've had wheezing before when the air is cold, but not this bad."
-He borrowed his friend's inhaler, and that helped some.
-He denies fevers, chills, nausea, or vomiting.
-The patient is high school student, and felt well until today, but notes "I've never had this much trouble breathing" (i.e. *severe problems with performing daily activities).*

Family History:
-Father has COPD, mother has atopic dermatitis.

Past Medical History:
-Reactive airways disease

Social History:
-Non-smoker, no alcohol, no recreational drugs.

Allergies: NKDA

<u>VIGNETTE 55</u>
Priapism

Demographics:
-Male, Age 47

Presenting Complaint / Initial user input:
-“My erection won’t go away.”

History, on open questioning:
-The patient notes 7 hours of a constant erection.
-He was “just sitting there at work when I noticed.”
-The patient was too embarrassed to stand up or tell anyone, so he worked a whole day before presenting.
-He has developed significant pain in his penis.
-He is also having trouble urinating now.

If asked directly:
-He denies any sexual arousal before these symptoms.
-He denies penile discharge, dysuria, bloody urine.
-No recent fevers, chills, or scrotal pain.
-The patient is a software programmer and has felt well until today, but “this came out of nowhere, and now I can’t think of anything else” (i.e. *some problems with performing daily activities).*

Family History:
-Father had a coronary artery disease, mother had a stroke.

Past Medical History:
-Sickle Cell Disease
-Diabetes (type II)
-Hypertension
-Depression

Social History:
- Active 2 pack/day smoker, occasional alcohol, occasional marijuana and cocaine.

Allergies: NKDA

<u>VIGNETTE 56</u>
Optic Neuritis

Demographics:
-Female, Age 31

Presenting Complaint / Initial user input:
-“I’m having eye pain.”

History, on open questioning:
-The patient notes pain in her left eye over the last 2 days.
-It started as a dull ache, but now it feels more “nagging and severe.”
-The pain is much worse when she looks to the side.
-She has also developed blurry vision in the left side “on the outside of my vision, I can only really see half of your face.”
-Today, the color of her bright pink shirt “seems kind of dull in some way” to her.
-She also notes that she “flashy lights when I was falling asleep last night” as well.

If asked directly:
-She endorses intermittent tingling in her legs recently.
-Last week, she had an “electric shock feeling when I bent over to tie my shoes.”
-She denies headache, neck pain, fever or chills.
-She denies any recent eye injury or redness.
-The patient is a fighter pilot, has otherwise felt well until 2 days ago, but “I can’t fly if I can’t see” (i.e. *severe problems with performing daily activities).*

Family History:
-Father had diabetes, mother has multiple sclerosis.

Past Medical History:
-No relevant PMH

Social History:
-Active 1 pack/day smoker, no alcohol, no recreational drugs.

Allergies: NKDA

<u>**VIGNETTE 57**</u>
Stable Angina

Demographics:
-Male, Age 48

Presenting Complaint / Initial user input:
-“I get chest pressure sometimes.”

History, on open questioning:
-The patient reports a few months of intermittent pressure in his chest.
-It only happens with exertion, and lately his noticed it when skipping the elevator and climbing the stairs at work.
-It is more of a pressure than a pain, “like a weight on my chest.”
-This discomfort is across his chest, and is sometimes associated with mild shortness of breath as well.
-It resolves on its own with rest.

If asked directly:
- Sometimes the discomfort is associated with pain in the left arm.
- He thought it was just indigestion because it feels similar.
- Each episode is just like the last one.
-He denies associated nausea, sweating, or lightheadedness.
-No recent travel, calf pain, or lower extremity swelling.
-No recent cough, fever, or diarrhea.
-The patient is novelist, and used to go for long walks on the beach, but “I can’t really do that anymore with feeling uncomfortable” (i.e. *some problems with performing daily activities).*

Family History:
-Father has diabetes and coronary artery disease.
-Mother died of a heart attack.

Past Medical History:
-Diabetes
-Hypertension
-Hyperlipidemia

Social History:
-Active 1 pack/day smoker, 1-2 alcoholic drinks per day. No recreational drugs.

Allergies: NKDA

VIGNETTE 58
Trigger finger

Demographics:
-Female, Age 40

Presenting Complaint / Initial user input:
-“My finger is locking.”

History, on open questioning:
-Gradual onset starting 1 month ago
-Pain and “catching” when bending finger
-Popping or clicking sensation when bending finger
-Finger gets stuck in a bent position, straightens with a snap
-Sensation of the finger “going out of joint”
-Swelling or stiffness in the finger, particularly in the morning

If asked directly:
-No obvious injury or trauma to the finger
-Wakes up with finger locked in the palm, although the finger gradually unlocks during the day
-Has a painful “nodule” in the palm at the base of the affected finger
-Work requires repetitive hand use and gripping actions
-Had surgery for carpal tunnel syndrome 1 month ago

Duration of symptoms:
-1 month

Activities of daily living:
-Some problems performing daily activities

Family history:
-Father has diabetes mellitus, type 2
-Mother has rheumatoid arthritis

Past medical history:
-Diabetes mellitus, type 2
-Carpal tunnel syndrome status-post surgery
-De Quervain’s tenosynovitis
-No smoking, alcohol, or recreational drugs

Allergies: NKDA

<u>VIGNETTE 59</u>
Sepsis

Demographics:
-Female, Age 84

Presenting Complaint / Initial user input:
-“I feel awful.”

History, on open questioning:
-The patient reports 5 days of worsening malaise and fatigue.
-She first noted “a little bit of pain with peeing”, and a foul odor to her urine.
-She then developed a fever to 39 degrees, nausea, and vomiting.
-She has developed pain in both flanks, and “now everything hurts.”
-“I feel like I’ve been run over by a bus.”
-She also endorses lightheadedness with standing, and two episodes of “just passing out.”

If asked directly:
-She reports a racing heart, and “I feel like I’m breathing fast.”
-This morning, she couldn’t figure out how to make her morning coffee, and forgot her husband’s name.
-She hasn’t micturated at all over the last 24 hours, and “usually I get up all night to pee.”
-She has also noted a new, blotchy rash on her legs.
-She denies slurred speech, facial droop, tingling, numbness, or weakness in her extremities.
-The patient is retired surgeon, and felt well until the last 5 days, but notes “I’ve never felt this bad” (i.e. *unable to perform daily activities).*

Family History:
-Father had diabetes, mother had rheumatoid arthritis

Past Medical History:
-Diabetes
-Rheumatoid arthritis
-Ulcerative Colitis
-Frequent UTIs

Social History:
-Active 1 pack/day smoker, no alcohol, no recreational drugs.

Allergies: Ceftriaxone

<u>VIGNETTE 60</u>

Acute Lower Urinary Tract Infection

Demographics:
-Female, Age 18

Presenting Complaint / Initial user input:
-“I’m having pain when I pee.”

History, on open questioning:
-The patient reports 2 days of dysuria.
-She notes a “burning sensation when I urinate, and I’m going a lot more often.”
-She has also developed a crampy sensation in the middle of her lower abdomen.
-This morning, her urine was “pink and cloudy, and it smells really bad.”

If asked directly:
-She is endorsing an “urgent need to pee all the time.”
-She has recently started having intercourse, and uses condoms.
-She denies fevers, chill, nausea, or flank pain.
-Her pain is worse when she pushes “very low down” in the middle of her abdomen.
-The patient is high school student and has felt well until today, and these symptoms are “just annoying, like my boyfriend” (i.e. *no problems with performing daily activities).*

FH:
-No relevant FH

PMH:
-No relevant PMH

SH:
-Non-smoker, no alcohol, occasional marijuana.

Allergies: Sulfonamides

<u>**VIGNETTE 61**</u>
Fracture of Foot

Demographics:
-Female, Age 46

Presenting Complaint / Initial user input:
-“I’m having foot pain.”

History, on open questioning:
-The patient reports 1 day of foot pain.
-The pain started when she turned her foot while stepping up on a curb.
-She notes she “turned it sideways, then felt immediate pain.
-The pain “one the outside of my foot.”
-The pain is sharp and severe, and worse with bearing weight.
-She has been unable to walk without assistance today.

If asked directly:
-The pain is much worse on the “outside” (lateral side) of her foot.
-It is very tender to touch the side of her foot, especially just below the ankle.
-She has developed swelling and bruising of the area.
-She denies pain in her ankle, or other injuries.
-The patient is state senator, and has “been hobbling around for the last day, but I can’t stop for something like this” (i.e. *some problems with performing daily activities).*

Family History:
-No relevant Family history

Past Medical History:
-Gastroesophageal reflux disease
-Ankle surgery

Social History:
-Active 1 pack/day smoker, no alcohol, no recreational drugs.

Allergies: Ibuprofen

VIGNETTE 63
Diverticulitis

Demographics:
- Male, Age 55

Presenting Complaint / Initial user input:
"I'm having new abdominal pain"

History, on open questioning:
- Pain started a few days ago and has been progressively getting worse
- Pain located predominantly in the lower abdomen on the left side
- Pain is constant
- Pain started as a dull 3/10 pain
- Today pain is more severe with cramping, 7/10 pain

Symptoms and risk factors, if asked directly:
- Chronic constipation issues
- Has had few episodes of transient left lower abdominal pain in the past few years that resolved in a few hours and were self limiting
- Worsening constipation last 1-2 days but still passing gas
- Had some new rectal bleeding yesterday, no rectal bleeding currently
- Has nausea and decreased appetite
- No vomiting
- Has felt slightly feverish today
- No dysuria, hematuria, flank pain
- No prior abdominal surgery
- No recent travel or exotic food ingestion

Duration of symptoms:
Days

Activities of daily living:
Severe problems performing daily activities

Family history: No relevant FH

Past Medical History:
Elevated BMI (30)

Past Social History:
non-smoker, does not drink alcohol, no other drug use history

Drug History:
Lisinopril

Allergies:
NKDA

<u>VIGNETTE 64</u>
Rupture of Achilles Tendon

Demographics:
-Male, Age 34

Presenting Complaint / Initial user input:
-“I’m having leg pain.”

History, on open questioning:
-The patient reports 30 minutes of leg pain.
-The pain started suddenly when he jumped up for a rebound while playing basketball.
-He notes “it was liked someone kicked me in the calf.”
-The patient heard a “snap”, then felt immediate pain.
-The pain is sharp and severe, and located at the lower calf.
-He is unable to walk.

If asked directly:
-It is very tender to touch the area just above his heel, in the back.
-He has developed significant swelling and bruising of the area.
-The pain is much worse with trying to stand up.
-He denies pain in her foot, knee, or other injuries.
-The patient is a retired professional basketball player, and has otherwise been well until the last 30 minutes, but now “can’t walk even a little” (i.e. *severe problems with performing daily activities).*

Family History:
-Mother has mixed connective tissue disorder, Father has Marfan’s disease.

Past Medical History:
-Obesity
-Biceps tendon rupture as a child

Social History:
-Non-smoker, drinks 1-2 beers per day, occasional cocaine.

Allergies: Lidocaine

VIGNETTE 65
Transient cerebral ischemia

Demographics:
Male, age 54

Presenting Complaint / Initial user input:
-"I've had a dizzy spell."

History, on open questioning:
This morning I became dizzy and found it difficult getting my balance. It felt as if my head was spinning round. I also noticed that my speech became slurred.

If asked directly:
This came on suddenly, and I have never had any similar symptoms before. I vomited once. I have no headache, visual disturbance, hearing problems, weakness or numbness. The symptoms lasted about 30 minutes and I now feel much better.

Duration of symptoms:
Hours

Activities of daily living:
No problems performing daily activities

Family History:
None

Past Medical History:
I have no past medical or surgical history and have been previously fit and well.

Social History:
Teacher

Allergies:
None

VIGNETTE 66
Small Bowel Obstruction

Demographics:
-Female, Age 58

Presenting Complaint / Initial user input:
-“I’m having abdominal pain.”

History, on open questioning:
-The patient reports 1 day of worsening diffuse abdominal pain.
-The pain started as a crampy discomfort, but now comes in “sharp waves.”
-She notes it is a “gassy pain”, but “the worst gas I’ve ever had.”
-She had developed significant nausea and multiple episodes of vomiting as well.
-She also notes abdominal distension “I look like I’m pregnant but I can’t be!”

If asked directly:
-Her pain is worse with pushing in her upper abdomen.
-It “sounds like a drum” when she taps on it.
-She is not passing gas (flatus).
-It did get a little better immediately after vomiting.
-Over the last few hours her vomiting has become incredibly foul smelling (feculent).
-She denies diarrhea, fevers, or chills.
-The patient is musician, and has otherwise been well until the last day, but now “can’t really do anything because it hurts so much when I stand up” (i.e. *severe problems with performing daily activities).*

FH:
-No relevant family history

PMH:
-S/p appendectomy and cholecystectomy
-S/p hysterectomy
-Inguinal hernia

SH:
-Non-smoker, no alcohol, occasional marijuana.

Allergies: Sulfonamides

<u>**VIGNETTE 67**</u>
Migraine

Demographics:
Female, Age 24

Presenting Complaint:
"I've had problems with my vision"

History, on open questioning:
Lost the sight in my right eye for about 15 minutes three days ago.
My vision was blurred as if I was looking through dirty water.
I had some zigzag lines in my right eye for about two minutes when this was happening.
I'm really worried.

If asked directly:
About 6 weeks ago, my right side went weak on two separate occasions. It lasted for about 30 minutes each time.
No headaches
I work in front of a computer and wear glasses. My last eye test was 6 months ago

FH:
-No relevant family history

PMH:
No previous headaches
No migraine
Appendix removed aged 10

Duration of symptoms:
hours

Activities of daily living:
No problems performing daily activities

SH:
Works as a secretary
Exercise frequently and play tennis twice a week.
Non smoker
Social drinking in moderation at the weekend.

Drug History: Microgynon 30 (levonorgestrel 150 micrograms, ethinylestradiol 30 micrograms)
Commenced six months ago

Allergies: None

<u>**VIGNETTE 68**</u>

Acute Angle-Closure Glaucoma

Demographics:
-Female, Age 72

Presenting Complaint / Initial user input:
-“My eye hurts and my vision is blurry.”

History, on open questioning:
-The patient notes sudden left eye pain 20 minutes ago.
-She notes she was the movie theatre, and suddenly her left eye became very painful.
-She then developed blurry vision, and when she stepped outside “the lights had halos around them.”
-She was unable to drive to the hospital, and called an ambulance.
-Since onset, she has developed nausea and one episode of emesis.

If asked directly:
-Her right eye vision is intact.
-The EMT told her that her eye left pupil was dilated.
-She has developed a headache now, and profuse tearing in her left eye.
-She denies any eye pain or injury, and it does not hurt to blink.
-She denies slurred speech, facial droop, tingling, numbness, or weakness.
-Patient is a film critic, and has felt well until today, but “it hurt so much I had to leave just before the climax—so frustrating!” (i.e. *some problems with performing daily activities).*

Family History:
-Father had glaucoma, mother had a stroke.

Past Medical History:
-Diabetes
-Hypertension
-COPD
-Migraines

Social History:
- Active 1 pack/day smoker, does not drink alcohol, no recreational drugs.

Allergies: Shellfish

<u>VIGNETTE 69</u>

Rotator cuff syndrome

Demographics:
-Male, Age 50

Presenting Complaint / Initial user input:
-“My shoulder hurts.”

History, on open questioning:
-Gradual onset starting 2 months ago
-Dull ache deep inside the shoulder
-Difficult to raise arm overhead or reach behind the back
-Pain worsens with repetitive overhead activity, lifting, and throwing

Symptoms and risk factors, if asked directly:
-No obvious injury or trauma to the shoulder
-Pain disturbs sleep, particularly when lying on the affected shoulder
-Arm on the affected side feels weaker
-Likes to play baseball and tennis
-Works as a painter or carpenter

Duration of symptoms:
-2 months

Activities of daily living:
-Moderate problems performing daily activities

Family history:
-Father was an athlete and had a rotator cuff tear
-Mother had diabetes mellitus (type 2) and frozen shoulder

Past medical history:
-None significant
-Social alcohol user
-No smoking or recreational drugs

Allergies:
-NKDA

VIGNETTE 70
Human immunodeficiency virus infection

Demographics:
Male, Age 29

Presenting Complaint / Initial user input:
Bad cough bringing up white sputum for a week. Fever on and off, shortness of breath.

History, on open questioning:
I've not been feeling great for the past few months. I've been feeling more and more tired with a poor appetite and had been losing weight. I have not been dieting and was happy to being with about losing weight but now I am worried. Shortness of breath initially only on walking up hill for example but has now progressed and I feels short of breath at rest or just with talking.

Symptoms and risk factors, if asked directly:
No stomach ache, no diarrhea
No blood in urine or stool
No pain anywhere really
I smoke 15 – 20 cigarettes a day.
I drink alcohol now and then – mostly at the weekends.
I don't take drugs now but in my late teens I did take a variety of illicit drugs. On occasions I injected, sharing needles with a group of friends. I don't know if any previous partners or friends have had any serious illness such as HIV.

Duration of symptoms:
Months

Activities of daily living:
Some problems performing daily activities

Family history:
I live with my partner and have no children.
I have had 2 previous sexual partners.

Past medical history:
nil

Allergies:
nil

<u>VIGNETTE 71</u>
Manic Episode

Demographics:
-Male, Age 32

Presenting Complaint / Initial user input:
-“I just found out I’m Jesus Christ.”

History, on open questioning:
-The patient reports he is “really excited about saving the world.”
-He endorses that he “found out I’m Jesus Christ a couple days ago”, and has been “staying up all night saving people with my Ipad.”
-He has “also been making millions on the side, but I’m just going to donate it all.”
-He is “frustrated with all the slower people in the world...if everyone was like me there wouldn’t be any more problems!”
-He does admit to feeling jumpy, and wired, “like when I used to use crystal meth.”

If asked directly:
-He has been distractible and irresponsible recently.
-He has had racing thoughts, “but I’m not worried about them, I’m a genius.”
-He has been going on spending sprees, “yeah, I just bought a Jaguar, but Jesus should drive a Jaguar.”
-He has had increased libido and has been “spending a lot of time trying to find my Mary, if you know what I mean.”
-He denies suicidal or homicidal ideation, visual or auditory hallucinations.
-He denies recent fevers, chills, or other somatic symptoms.
-The patient reports he is also the President of the United States, “No seriously, anyone can be President these days, why not Jesus?” (i.e. *some problems with performing daily activities).*

FH:
-No relevant FH

PMH:
-No relevant PMH

SH:
-Non-smoker, occasional alcohol, occasional marijuana and cocaine.

Allergies: “I’m allergic to pagans.”

VIGNETTE 72
Kidney Stone

Demographics:
-Female, Age 33

Presenting Complaint / Initial user input:
-"I'm having severe pain in my side."

History, on open questioning:
-The patient reports 3 hours of left sided flank pain.
-She notes an intermittent, sharp, stabbing pain in her L flank.
-"It was bad at first, but now it's killing me!"
-She reports that she "felt every bump" on the way in today.
-Her pain also "shoots down into my groin" at times.
-She has also noted a pinkish tinge to her urine.

If asked directly:
-Her pain is worse with movement and pushing just below her ribs.
-She reports nausea, and two episodes of vomiting.
-Her pain comes in waves and fluctuates in intensity.
-She has only been urinating in small amounts over the last 2 days, and it has been dark yellow.
-The patient is currently fasting for Ramadan, and endorses decreased intake of food and fluids.
-She denies fevers, chills, diarrhea, or trauma to her flank.
-The patient is farmer and has been "outdoors all day harvesting for the last week, but I can barely move with this pain" (i.e. *severe problems with performing daily activities).*

Family History:
-Father had kidney stones, mother had cholelithiasis.

Past Medical History:
-Hyperparathyroidism
-Obesity
-S/P Gastric Bypass

Social History:
-Non-smoker, no alcohol, no recreational drugs.

Allergies: Morphine

<u>VIGNETTE 73</u>
Hypertrophic cardiomyopathy

Demographics:
25 year old female, corporate worker

Presenting Complaint / Initial user input:
A few weeks ago, it was a hot day I had been running on a particular hard terrain. After stopping, I started feeling tired, unwell with darkness in front of my eyes and fell to the floor. I was told the colour drained from my face and I regained consciousness within a few minutes and walked home. Went to A and E and all was fine. Went for a run today and the same thing happened.

History, on open questioning:
Enjoys outdoor activities and goes running for 5-6 miles, 2-3 times a week.
No incontinence, no tongue biting.
Quick recovery with no post-episode drowsiness or confusion

Symptoms and risk factors, if asked directly:
Never smoked, alcohol 4 units/ week

Duration of symptoms:
A few minutes
A similar episode happened two years ago.

Activities of daily living:
No problems performing daily activities

Family history:
Parents are alive and well. Has a younger sister, who is also well. No sudden unexpected death in the family. No medical issues.

Past Medical History:
During my teens, I used to pass out in hot or closed environment. Clinical examination at the time was unremarkable.

Drug History:
none

Allergies:
None

<u>VIGNETTE 74</u>
Ectopic Pregnancy

Demographics:
-Female, Age 36

Presenting Complaint / Initial user input:
-“I’m having vaginal bleeding.”

History, on open questioning:
-The patient reports 4 hours of increasing vaginal bleeding.
-She notes she has been “trying to get pregnant” but hasn’t been successful.
-Her last menstrual period was 5 weeks ago, but this bleeding is “much heavier.”
-She is “using 2 pads per hour, and there are clots.”
-She also notes right-sided suprapubic pain intermittently this morning.
-It is sharp and stabbing, but intermittent.

If asked directly:
-The bleeding started suddenly and is “nothing like my period.”
-Her pain is worse when she pushes “very low down” on her right abdomen.
-She has developed lightheadedness and a “feeling like I’m going to pass out.”
-She also notes nausea and a feeling of general malaise.
-She is taking “herbal supplements to help me get pregnant.”
-She denies pain with urination, recent fever or chills.
-The patient is yoga instructor and has felt well until today, but now “can’t stand up without feeling dizzy” (i.e. *some problems with performing daily activities).*

FH:
-No relevant FH

PMH:
-Pelvic Inflammatory Disease
-Chlamydia
-S/p appendectomy

SH:
-Prior 1 pack per day smoker, no alcohol, occasional marijuana.

Allergies: PCN

<u>**VIGNETTE 75**</u>

Deep Vein Thrombosis of the Lower Extremity

Demographics:
-Male, Age 65

Presenting Complaint / Initial user input:
-“I’m having calf pain and swelling.”

History, on open questioning:
-The discomfort started 2 days ago in the back of the right calf.
-It started as a mild pulling sensation, “like I strained it.”
-Located in the upper calf, and worsening over the last day.
-The pain is worse with walking.
-There has been increasing swelling in his foot and ankle.

If asked directly:
-Also noting a spot of pain in the upper calf that is “tender and hard”.
-No chest pain, shortness of breath, nausea, sweating, or lightheadedness.
-The swelling is “soft and squishy in my ankle.”
-The patient just returned from a flight to Australia 3 days ago.
-No recent cough, fever, or diarrhea.
-Patient is a business executive, and usually exercises daily without pain (i.e. *no problems with performing daily activities),*
-However over the last 2 days he has been unable to run on his treadmill due to pain (i.e. *some problems with performing daily activities).*

Family History:
-Mother has hypertension.
-Father had a pulmonary embolus and is on warfarin.

Past Medical History:
-Diabetes (Type II)
-Hypertension

Social History:
-Active 1 pack/day smoker, 1-2 alcoholic drinks per day. No recreational drugs.

Allergies: NKDA

<u>VIGNETTE 76</u>
Dislocation of Shoulder Joint

Demographics:
-Male, Age 20

Presenting Complaint / Initial user input:
-“I’m having shoulder pain.”

History, on open questioning:
-The patient reports 1 hour of right shoulder pain.
-The pain started while he was spiking a ball playing volleyball.
-He notes he “felt a pop”, and had immediate sharp pain.
-The pain is severe, and worse with any movement of the shoulder.

If asked directly:
-He has some numbness and weakness in his arm as well.
-His had noted a “divot” at the top of his shoulder.
-He denies elbow, wrist, or hand pain.
-He notes multiple prior injuries to his shoulder.
-The patient is construction worker, and has otherwise been well until today, but now “can’t move my shoulder at all” (i.e. *some problems with performing daily activities).*

FH:
-No relevant FH.

PMH:
-Prior rotator cuff injury
-S/p arthroscopy of right shoulder

SH:
-Non-smoker, drinks 1-2 beers per day, no recreational drugs.

Allergies: Ibuprofen

<u>VIGNETTE 77</u>
Viral Conjunctivitis

Demographics:
32yr, Male

Presenting Complaint / Initial user input:
- “both my eyes are red, sore and gunky”

Duration of symptoms: (few) days

History, on open questioning:
- Initially, the right eye was red and a bit sore.
- Then it started to feel dry and have some discharge coming from it
- Then just yesterday the other eye started to become red and have discharge too
- That’s when I came to see the doctor

Symptoms and risk factors, if asked directly:
- He has no loss of vision, or flashing lights or floaters
- He does not wear contact lenses
- He does not have any fever
- “it feels more sore than it does painful”
- He did have a runny nose and sore throat a few days prior to getting the eye symptoms
- He does not have any allergies

Activities of daily living:
- No problems performing daily activities.
- Works as a gardener and able to work as long as he wears protective glasses. He wondered if something may have gone into his eye when working but can`t remember a specific incident.

Family history: Father is type 2 diabetic.

Past Medical History: No medical history of note.

Drug History: Smokes 5/day, drinks “socially”. No other recreational or prescribed drugs.

Allergies: No known allergies.

VIGNETTE 78
Lower Gastrointestinal Bleed

Demographics:
-Female, Age 81

Presenting Complaint / Initial user input:
-“I’m bleeding from below.”

History, on open questioning:
-The patient reports 2 days of intermittent bloody stools.
-She notes that yesterday she saw “a few flecks” around her stool.
-Today, her stool is “bright red.”
-“The whole toilet was red today.”
-She is also endorsing urgency to stool (tenesmus).
-She has also started to feel a little lightheaded.
-She denies abdominal pain.

If asked directly:
-She denies nausea or vomiting.
-She denies “throwing up blood.”
-Her stool is “brown underneath, red on top.”
-She denies black stool.
-She also notes some general malaise, but no chest pain or shortness of breath.
-She denies diarrhea, fever, or chills.
-The patient is retired chef, and has otherwise been well until the today, but now “I don’t want to leave the house” (i.e. *some problems with performing daily activities).*

Family History:
-Father had diverticulitis, mother died of a heart attack.

Past Medical History:
-Diabetes
-Hypertension
-Hyperlipidemia
-Peripheral vascular disease
-Von Willebrand’s Disease

Social History:
-Active 1 pack per day smoker, drinks occasionally, no recreational drugs.

Allergies: NKDA

<u>VIGNETTE 79</u>
Trigeminal Neuralgia

Demographics:
-Female, Age 62

Presenting Complaint / Initial user input:
-“I have pain in my face.”

History, on open questioning:
-The patient notes intermittent facial pain over the last week.
-She endorses “episodes of severe, shooting pain in the right side of my face.”
-The pain lasts less than a minute, but is “like someone stuck me with a hot poker.”
-Sometimes, these episodes are triggered by chewing gum or brushing her teeth.
-“It’s crazy, nothing helps this pain, and it’s the worst I’ve ever had.”

If asked directly:
-She notes she feels fine otherwise, and the “electric shock is only in my face.”
-The pain is worse with even very light touch on her cheek.
-Her “attacks” have become more frequent and intense over the last week.
-She denies other headache, visual changes, slurred speech, or facial droop.
-She denies tingling, numbness, or weakness.
-No recent fevers, chills, or trauma to the face.
--The patient is biophysics professor and has felt well until these last week, but these symptoms “sometimes hurt to even speak” (i.e. *some problems with performing daily activities).*

Family History:
-Mother had a stroke, father had polymyalgia rheumatica.

Past Medical History:
-Diabetes
-Multiple Sclerosis

Social History:
- Active 1 pack/day smoker, no alcohol, no recreational drugs.

Allergies: Neurontin

<u>**VIGNETTE 80**</u>

Plantar Fasciitis

Demographics:
-Male, Age 16

Presenting Complaint / Initial user input:
-"I'm having foot pain."

History, on open questioning:
-The patient reports 2 days of foot pain.
-The pain started gradually over the last two days, and is only on the bottom of his right foot.
-He notes he went for a run 3 days ago, and "stepped on a tree root".
-The patient felt immediate sharp pain, but it dissipated and he was able to finish his run.
-The pain is now worse with bearing weight, but he is able to walk on it.

If asked directly:
-He is exquisitely tender on the bottom of his foot.
-It is especially tender just in front of his heel.
-He has developed mild swelling of the area, but no bruising.
-He denies pain in his ankle, or other injuries.
-The patient is high school student, and notes he can "still do stuff, but man it hurts!" (i.e. *no problems with performing daily activities).*

Family History:
-Mother has rheumatoid arthritis, father has ankylosing spondylitis.

Past Medical History:
-Appendectomy

Social History:
-Non-smoker, no alcohol, no recreational drugs.

Allergies: NKDA

VIGNETTE 81
Pneumonia

Demographics:
-Male, Age 24

Presenting Complaint / Initial user input:
-"I can't stop coughing."

History, on open questioning:
-The patient reports 3 days of a worsening cough.
-He first noticed a dry, nagging, cough, but "now I'm coughing all the time."
-The cough has become productive of rusty-colored sputum.
-He also notes shaking chills last night, and fever to 38 degrees on a home thermometer.
-Today, he has developed shortness of breath as well.
-"The cough kept me up all night, and my lungs just keep rattling."

If asked directly:
-He reports diffuse chest pain with coughing and taking a deep breath.
-He has also developed generalized malaise and fatigue.
-His cough has not improved with over-the-counter medications.
-He endorses decreased appetite and nausea as well.
-He denies recent travel, calf pain, or lower extremity swelling.
-The patient works in retail, and felt well until 3 days ago, but notes "my boss sent me home today because she said I sounded horrible" (i.e. *some problems with performing daily activities).*

Family History:
-Father has COPD, mother has asthma

Past Medical History:
-Cystic Fibrosis
-Diabetes
-Gastroesophageal reflux disease

Social History:
-Non-smoker, no alcohol, no recreational drugs.

Allergies: PCN

<u>VIGNETTE 82</u>
Meniere's Disease

Demographics:
-Female, Age 32

Presenting Complaint / Initial user input:
-"I feel dizzy."

History, on open questioning:
-The patient reports 2 hours of dizziness.
-She describes the dizziness as "the world spinning around me."
-The symptoms came on suddenly, without warning.
-She has also noticed a ringing sound in her right ear, "like a buzzing."
-She the developed intermittent nausea and one episode of vomiting.

If asked directly:
-She has had symptoms like this intermittently for months, but today they haven't gone away.
-When pressed, she does endorse a sensation of fullness in her right ear "I thought it was just wax."
-She notes hearing loss in her right ear as well.
-She denies headache at any time.
-She denies double vision, tingling, numbness, or weakness.
-She denies recent illness, fevers, or chills.
-The patient is a step-aerobics instructor and has felt well until today, but today she "couldn't even try the steps" (i.e. *some problems with performing daily activities).*

FH:
-Father has HTN, mother has "vertigo."

PMH:
-Migraines
-Multiple ear infections as a child
-Asthma

SH:
-Non-smoker, occasional alcohol, no recreational drugs.

Allergies: NKDA

VIGNETTE 83
Trigger Finger

Demographics:
-Female, Age 28

Presenting Complaint / Initial user input:
-“I’m having finger pain.”

History, on open questioning:
-The patient reports 2 weeks of right index finger pain.
-She notes the pain has worsened gradually over the past 2 weeks.
-It is particularly “nagging” in the morning, and worse with flexing her finger.
-She endorses “a stiffness” in his finger, and sometimes a “clicking sound.”

If asked directly:
-She has tenderness and a small bump at the base of her index finger.
-Her finger “locks up sometimes when I flex it.”
-It is particularly bad when she uses a power drill.
-She denies redness, fever, or chills.
-She denies other hand pain, or wrist pain.
-The patient is carpenter, and has otherwise been well until the last two weeks, but now is “having trouble using my tools” (i.e. *some problems with performing daily activities).*

FH:
-No relevant family history.

PMH:
-Diabetes
-Prior De Quervain's tenosynovitis

SH:
-Active 1 pack per day smoker, occasional alcohol, no recreational drugs.

Allergies: Sulfonamides

<u>VIGNETTE 84</u>

Depressive Disorder

Demographics:
-Female, Age 56

Presenting Complaint / Initial user input:
-"I'm feeling really sad."

History, on open questioning:
- The patient reports she has had worsening feelings of hopelessness and depression over the last week.
-She has been "crying all day" for the last two days.
-She also notes difficulty sleeping, and "didn't sleep at all last night."
-She notes that she also feels increasingly guilty "about being useless."
-Today she has been "thinking more about death", but she denies a plan to kill herself.

If asked directly:
-She has had increasing difficulty concentrating over the last week.
-Her energy level has been much lower, "I'm just slower at things."
-She also notes deceased appetite and is eating less.
-She denies homicidal ideation, visual or auditory hallucinations.
-She denies recent fevers, chills, or other somatic symptoms.
-The patient is a therapist and has tried to manage these symptoms at home "but I'm not really interested in anything these days" (i.e. *some problems with performing daily activities).*

Family History:
-No relevant FH

Past Medical History:
-No relevant PMH

Social History:
-Non-smoker, occasional alcohol, occasional marijuana.

Allergies: NKDA

<u>VIGNETTE 85</u>
Hodgkin’s Lymphoma

Demographics:
Male, age 20

Presenting Complaint / Initial user input:
“I have a lump on the side of my neck.”

History, on open questioning:
He noticed the lump on his neck about 6 months ago and it has been getting bigger.
The lump is not painful.
He feels tired most days.

Symptoms and risk factors, if asked directly:
He does not have cold symptoms such as a sore throat.
He is not aware of any other lumps on his body.
He does seem to sweat more at night.
He has had a low grade fever these past couple of weeks.
He may have lost a few pounds this past year.
He denies ever having chemotherapy or radiation treatment.
He has been sexually active with one female in the past.
He has never been tested for HIV.
He doesn’t have joint pains, but does have discomfort in his lower back whenever he lies flat on his back.

Duration of symptoms:
Has been feeling under the weather for months now.

Activities of daily living:
He is able to do his ADLs and attend school, but is having a hard time keeping up with his homework.

Family history:
No relevant family history.

Past Medical History:
history of infectious mononucleosis as a teenager

Drug History:
Has never smoked. He used to drink about 5 beers a week, but stopped recently due to pain all over his body when he drinks. He has used cocaine and marijuana; he last used about 2 years ago.

Allergies: NKDA

<u>VIGNETTE 86</u>
Bacterial arthritis

Demographics:
- Female, Age 45

Presenting Complaint / Initial user input:
“I’m having new onset in my right knee”

History, on open questioning:
- Pain started yesterday and has been progressively getting worse
- Pain located throughout the whole right knee
- Right knee is red, hot and swollen
- Severe (9/10) and sharp pain
- Knee is painful to touch

Symptoms and risk factors, if asked directly:
- Barely able to move the knee due to the pain, limited range of motion
- No recent trauma or injury to R knee
- Currently with no joint pain apart from the R knee
- Subjective fevers and chills for the last day
- Took some Tylenol which helped slightly with pain and fever but no significant improvement
- No new rashes
- No recent abdominal pain, diarrhea
- No recent travel or calf pain
- No recent tick bite
- No history of arthritis in the past
- History of Crohn’s disease on immunosuppression (adalimumab)
- No history of DVT/PE
- No history IV drug use
- Married for 10 years with 2 children (no new sexual partners)

Duration of symptoms:
days

Activities of daily living:
Severe problems performing daily activities

Family history:
No relevant FH

Past Medical History:
Crohns disease

Past Social History:
non-smoker, does not drink alcohol, no other drug use history

Drug History:
Adalimumab

Allergies: NKDA

<u>VIGNETTE 87</u>
Sprain of Ankle

Demographics:
-Female, Age 44

Presenting Complaint / Initial user input:
-"I'm having ankle pain."

History, on open questioning:
-The patient reports 3 hours of ankle pain.
-The pain started when she turned her ankle while dancing in high heels.
-She notes she "stepped on my partner's foot and turned it."
-The patient felt immediate sharp pain.
-The pain is worse with bearing weight, but she is able to walk on it.

If asked directly:
-The pain is a little worse on the "outside" (lateral side) of her ankle.
-It is not tender to touch the "outside bump" (lateral malleolus).
-She has developed swelling of the area.
-She denies pain in her foot, or other injuries.
-The patient is dance teacher, and notes she can "get by OK" with this pain (i.e. *no problems with performing daily activities).*

FH:
-Mother has osteoporosis, Father has hypertension.

PMH:
-No relevant PMH

SH:
-Active 1 pack/day smoker, no alcohol, no recreational drugs.

Allergies: NKDA

<u>VIGNETTE 88</u>
Diverticulitis

Demographics:
-Male, Age 62

Presenting Complaint / Initial user input:
-“I’m having abdominal pain.”

History, on open questioning:
-The patient reports 4 days of worsening abdominal pain.
-The pain initially started as a “mild discomfort which I thought was gas.”
-It is now “crampy and sharp sometimes”, and located in the left lower abdomen.
-He has had nausea, and a few episodes of vomiting as well.
-“The worst part is the diarrhea, it hurts and it’s bloody now.”

If asked directly:
-His pain is much worse if he pushes on the left, lower side of his abdomen.
-Today he has had a fever and shaking chills all day.
-He has had generalized malaise and loss of appetite as well.
-He denies upper abdominal pain.
-The patient is cricket coach, but hasn’t been able to leave the house because “I have to stay near the loo” (i.e. *some problems with performing daily activities).*

FH:
-Father has diverticulosis, mother had a stroke.

PMH:
-Diverticulosis
-HTN

SH:
-Non-smoker, drinks 1-2 beers per day, no recreational drugs.

Allergies: NKDA

<u>**VIGNETTE 89**</u>

Thyrotoxic Crisis

Demographics:
-Male, Age 32

Presenting Complaint / Initial user input:
-“I feel awful.”

History, on open questioning:
-The patient reports 4 hours of restlessness and palpitations.
-He first noticed a sense of anxiety this morning when he woke up, and he has been “irritable” all morning.
-He then noticed palpitations and a “racing heart”, and a “funny feeling” in his chest.
-Since then, he has developed “lots of sweating, even though my apartment is cold.”
-He then measured his temperature at 41 degrees on a home thermometer, and called 911.
-He also notes some lightheadedness, “I felt like I was going to pass out.”

If asked directly:
-He reports intermittent symptoms like this for months, but not as bad as today.
-He endorses some disorientation when trying to lock up the house.
-His notes generalized malaise and exhaustion “like I just ran a marathon.”
-He denies recent cough, dysuria, or rashes.
-He denies chest pain, recent travel, calf pain, or lower extremity swelling.
-He denies nausea, vomiting, or diarrhea.
-The patient is football coach, and felt well until this morning, but notes “I feel so badly I can barely move” (i.e. *severe problems with performing daily activities).*

Family History:
-No significant family history.

Past Medical History:
-Hyperthyroidism
-Recent pneumonia

Social History:
-Active 1 pack/day smoker, no alcohol, no recreational drugs.

Allergies: Penicillin

<u>**VIGNETTE 90**</u>
Appendicitis

Demographics:
- Male, Age 19

Presenting Complaint / Initial user input:
“I’m having new abdominal pain”

History, on open questioning:
- Pain started about 5 hours ago
- Pain located initially in the mid-abdomen around the belly button area but over the last couple hours has moved to the right lower portion of the abdomen
- Cramping type of abdominal pain with 8/10 intensity

Symptoms and risk factors, if asked directly:
- About 1 hour ago started to develop nausea and vomiting
- About 1 hour ago started to feel subjective fevers
- Some loose stools over last few hours but non-bloody
- No constipation
- No cough, shortness of breath, chest pain
- No dysuria, hematuria, flank pain
- No hematemesis
- No jaundice
- No prior abdominal surgery
- No recent travel or exotic food ingestion

Duration of symptoms: Hours

Activities of daily living: *Severe problems performing daily activities*

Family history:
No relevant family history

Past Medical History:
None

Past Social History:
non-smoker, does not drink alcohol, no other drug use history

Drug History:
None

Allergies:
NKDA

VIGNETTE 91
Diabetic Ketoacidosis

Demographics:
-Female, Age 19

Presenting Complaint / Initial user input:
-"I feel awful."

History, on open questioning:
-The patient reports 2 days of generalized malaise and fatigue.
-She first noticed that "felt really thirsty" yesterday, "but no matter how much I drink I still feel thirsty."
-Then she was "up all night peeing, like when I had a UTI."
-This morning, she developed nausea and crampy, diffuse abdominal pain.
-She also feels like her "heart is racing", and notes palpitations.
-Her boyfriend also told her that "my breath smells like nail polish remover."

If asked directly:
-She started running low on insulin last week, and has been taking less than her usual dose.
-She endorses facial flushing and lightheadedness.
-She has been unable to concentrate, and couldn't remember her address today.
-No recent cough, fever, chills, dysuria or hematuria.
-Patient is a high school student and has felt well until the last 2 days, but "I even had to miss school...I never miss school!" (i.e. *some problems with performing daily activities).*

Family History:
-Mother has diabetes, father has renal failure.

Past Medical History:
-Diabetes (Type I)
-Gastroesophageal reflux disease

Social History:
-Non smoker, no alcohol, occasional marijuana and Adderall.

Allergies: NKDA

<u>VIGNETTE 92</u>
Whiplash Injury to Neck

Demographics:
-Male, Age 24

Presenting Complaint / Initial user input:
-“I’m having neck pain.”

History, on open questioning:
-The patient reports 2 hours of neck pain.
-The pain started just after a motor vehicle collision in which he was “rear ended”.
-He was wearing his seat belt, and got out of the car at first without pain.
-He then developed a ‘sore’ sensation of dull pain in the “back and sides” of his neck (paraspinous) over the last 2 hours.
-He denies tingling or numbness anywhere.

If asked directly:
-The pain is only on the outsides, not “in the middle” (no midline pain).
-The pain is not worse with “pushing on the bones” (no midline tenderness).
-He denies tingling, numbness, or weakness in his extremities.
-He is able to walk, and sit up in a chair.
-He can turn his neck from side to side.
-He denies hitting his head, or other injuries.
-The patient is racecar driver, and notes “I’ve worked through worse pains” (i.e. *no problems with performing daily activities).*

FH:
-No relevant FH

PMH:
-Anxiety

SH:
-Non-smoker, drinks 3-4 beers per week, no recreational drugs.

Allergies: Ibuprofen

<u>VIGNETTE 93</u>
Bacterial Meningitis

Demographics:
-Male, Age 19

Presenting Complaint / Initial user input:
-“I’m having headache and neck pain.”

History, on open questioning:
-The patient reports 1 day of worsening headache and neck pain.
-The pain started overnight, and is now diffuse and severe.
-He then developed increasing neck pain, which is worse with moving his neck and bending forward.
-He also notes a subjective fever and shaking chills, “which makes my neck pain much worse”.
-He denies tingling, numbness, or weakness.

If asked directly:
-He reports that light and sound have become bothersome.
-He has also noted a new purple rash over the last few hours.
-His headache gets worse when he moves his head and neck.
-The pain is worse with pushing in the middle of the neck as well.
-He denies tingling, numbness, or weakness in his extremities.
-His roommate has been feeling sick as well, and has a fever.
-The patient is college student, and felt well until today, but notes “I’ve never felt this bad” (i.e. *unable to perform daily activities).*

FH:
-No relevant FH.

PMH:
-Diabetes

SH:
-Active 1 pack/day smoker, no alcohol, no recreational drugs.

Allergies: PCN

<u>**VIGNETTE 94**</u>
Cholecystitis

Demographics:
- Female, Age 45

Presenting Complaint / Initial user input:
"I'm having new abdominal pain"

History, on open questioning:
- Pain located mostly in the upper abdomen particularly on the right side
- Pain started yesterday night, initially with dull pain
- Pain has persisted and worsening over last few hours, now with constant cramping pain about 7/10 in severity

Symptoms and risk factors, if asked directly:
- Onset of pain occurred about one hour after a large meal during a dinner party
- Has nausea, vomiting
- Unable to eat or drink anything this morning due to nausea
- Has felt fevers and chills last few hours
- No constipation or diarrhea
- No cough, shortness of breath, chest pain
- No dysuria, hematuria, flank pain
- No rectal bleeding or hematemesis
- No jaundice
- No prior abdominal surgery
- No recent travel or exotic food ingestion

Duration of symptoms: Days

Activities of daily living:
Severe problems performing daily activities

Family history:
No relevant FH

Past Medical History:
Diabetes

Past Social History:
non-smoker, does not drink alcohol, no other drug use history

Drug History:
Metformin, OCPs

Allergies:
NKDA

VIGNETTE 95
Retinal Detachment

Demographics:
-Male, Age 64

Presenting Complaint / Initial user input:
-"I'm having visual changes."

History, on open questioning:
-The patient notes sudden appearance of visual changes in his right eye 90 minutes ago.
-He had just finished working out at the gym, then noticed "tiny flashes of light in my vision."
-These progressed to "wavy lines, and funny floating specks all over the place."
-He now notes that the right side of his vision is "blurry like when then try to blank someone's face out on TV."
-His eye does not hurt.

If asked directly:
-He denies any eye injury or redness.
-He denies headache, head trauma, or neck pain.
-His left eye vision is intact.
-He denies slurred speech, facial droop, tingling, numbness, or weakness.
-He notes that the he was feeling fine today before these symptoms.
-Patient is a machinist, and has felt well until today, but "I wouldn't dream of going back to work like this" (i.e. *some problems with performing daily activities).*

Family History:
-Father had a stroke, mother has diabetes and macular degeneration

Past Medical History:
-Diabetes
-Iritis
-S/p cataract surgery

Social History:
-Active 1 pack/day smoker, no alcohol, no recreational drugs.

Allergies: NKDA

<u>VIGNETTE 96</u>

Pneumothorax

Demographics:
-Male, Age 21

Presenting Complaint / Initial user input:
-"I'm having shortness of breath."

History, on open questioning:
-The patient reports 45 minutes of chest pain and shortness of breath.
-It started suddenly while swimming competitively today.
-He notes he was "half-way through my 1500 freestyle, and when I took a deep breath to turn I felt a sudden stabbing in my chest."
-He then developed severe shortness of breath, and "I had to get right out of the pool because I felt like I was going to pass out."

If asked directly:
-He reports a sharp, right-sided chest pain with breathing.
-He feels like his breaths are "cut short", and he "can't get any air in."
-He also notes a racing heart and lightheadedness.
-He denies recent wheezing or cough.
-He denies recent fevers, chills, calf pain, or lower extremity swelling.
-The patient is high school student, and felt well until today, but notes "this feels bad, I'm never this winded" (i.e. *severe problems with performing daily activities).*

Family History:
-Father has asthma, mother has COPD.

Past Medical History:
-Asthma
-S/p tube thoracostomy

Social History:
-Active 1 pack/day smoker, no alcohol, occasional marijuana.

Allergies: Sulfonamides

<u>**VIGNETTE 97**</u>

Fracture of Ankle

Demographics:
-Female, Age 24

Presenting Complaint / Initial user input:
-“I’m having ankle pain.”

History, on open questioning:
-The patient reports 30 minutes of ankle pain.
-The pain started when she turned her ankle while practicing parkour.
-She notes she “landed a flip funny and turned it.”
-The patient heard a “pop”, then felt immediate pain.
-The pain is sharp and severe, and worse with bearing weight.
-She is unable to walk.

If asked directly:
-The pain is much worse on the “outside” (lateral side) of her ankle.
-It is very tender to touch the “outside bump” (lateral malleolus), especially in the back.
-She has developed swelling and bruising of the area.
-She denies pain in her foot, or other injuries.
-The patient is student and parkour enthusiast, and has otherwise been well until the last 30 minutes, but now “can’t walk” (i.e. *some problems with performing daily activities).*

FH:
-Mother has osteoporosis, Father has hypertension.

PMH:
-No relevant PMH

SH:
-Active 1 pack/day smoker, no alcohol, no recreational drugs.

Allergies: NKDA

<u>VIGNETTE 98</u>
Tension-type Headache

Demographics:
-Female, Age 28

Presenting Complaint / Initial user input:
-“I have a headache.”

History, on open questioning:
-The patient reports 4 days of an intermittent headache.
-She notes the pain is “like a tight rubber band around my head.”
-It “starts in the back, then spreads all over.”
-She notes a moderate, diffuse headache, lasting “about an hour.”
-She also describes para-spinous neck pain after moving apartments last week.
-“Work has been really stressful lately, and it seems to make my headaches worse.”
-Her headache improves with paracetamol

If asked directly:
-She reports that the “muscles in the back of my neck are really tight, and it hurts to push on them.”
--She has had symptoms like this intermittently for months, but today they haven’t gone away.
-Her headache is not made worse by physical activity.
-She denies associated nausea or vomiting.
-She denies photophobia or phonophobia.
-She denies slurred speech, facial droop, tingling, numbness, or weakness.
-She denies recent illness, fevers, or chills.
-The patient is a sales executive and has been “able to work through the pain” (i.e. *some problems with performing daily activities).*

Family History:
-No relevant family history.

Past Medical History:
-Migraines
-Generalized Anxiety Disorder

Social History:
-Non-smoker, drinks 2-3 glasses of wine per day, no recreational drugs.

Allergies: NKDA

VIGNETTE 100
Diffuse Interstitial Pulmonary Fibrosis

Demographics:
Male, age 71

Presenting Complaint / Initial user input:
"I feel short of breath and my cough just won't go away."

History, on open questioning:
The patient has had a dry cough for several months.
He gets short of breath climbing stairs and walking around the neighborhood, which wasn't as much of a problem a year ago.
Nasal sprays and inhalers haven't helped much with his cough.

Symptoms and risk factors, if asked directly:
He denies chest pressure.
He denies fevers or joint pains
He does not have Raynaud's phenomenon.
He is not aware of any exposure to asbestos or silica.
He has never had chemotherapy or radiation treatment.
He does not own any pets or have exposure to birds or pigeons.
He denies problems with heartburn.
He worked in a metal shop for most of his adult life and has never worked on a farm.

Duration of symptoms:
The cough has been present for at least a year. The shortness of breath has been more noticeable these past 6 months, but he recalls feeling short of breath for at least the past 2 years.

Activities of daily living:
He can do his ADLs, but has to take breaks. Carrying groceries into the house is difficult due to the shortness of breath. Sometimes the cough affects his willingness to commit to social engagements.

Family history:
No family history of lung disease, including lung cancer.

Past Medical History:
BPH, hypertension, hyperlipidemia.

Drug History:
He has a 5-pk-year smoking history. Quit smoking 20 years ago

Allergies:
NKDA

VIGNETTE 102
Malignant tumor of testis

Demographics:
Male, age 35

Presenting Complaint / Initial user input:
I have a lump on my testicle

Duration of symptoms:
I noticed it in the shower yesterday

History, on open questioning:
I might have knocked a few days back playing football

Symptoms and risk factors, if asked directly:
It is on the actual testicle
It is not painful
I do check my testicles regularly and have never noticed it before
I do not have a fever
I am married and have had no other partners

Family history: Nil

Past Medical History: a number of sports injuries

Drug History: Nil

Allergies: NKDA

<u>VIGNETTE 103</u>
Rapid atrial fibrillation

Demographics:
Female, age 67

Presenting Complaint / Initial user input:
“I can’t breathe my heart is racing”

Duration of symptoms:
One hour

History, on open questioning:
Previous palpitations “I think my pulse has been a regular for a while”
Breathlessness “I feel short of breath even sitting down”

Symptoms and risk factors, if asked directly:
Pulse is rapid “My heart is thumping in my chest”

Family history:
Nil

Past Medical History:
heart-attack 10 years ago

Drug History:
isosorbide mononitrate 60mg aspirin 75

Allergies:
Nil

VIGNETTE 104
Adhesive capsulitis of shoulder

Demographics:
-Female, Age 50

Presenting Complaint / Initial user input:
-“I have a stiff shoulder.”

History, on open questioning:
-Gradual onset starting 3 months ago
-Limited range of motion in shoulder
-Any movement is moderately painful
-Generalized mild tenderness throughout the shoulder

Symptoms and risk factors, if asked directly:
-No obvious injury or trauma to shoulder
-Pain worsens at night, sometimes disrupting sleep
-Had a mastectomy on the same side of affected shoulder 3 months ago

Duration of symptoms:
-3 months

Activities of daily living:
-Moderate problems performing daily activities

Family history:
-Father has Parkinson’s disease
-Mother had a stroke

Past medical history:
-Diabetes mellitus, type 1 or 2 (especially type 1)
-Breast cancer status-post mastectomy
-Hypothyroidism
-Cervical disc herniation
-Prior rotator cuff injury
-No smoking, alcohol, or recreational drugs

Allergies:
-NKDA

<u>VIGNETTE 105</u>
Primary familial hypertrophic cardiomyopathy

Demographics:
Female, age 47

Presenting Complaint / Initial user input:
"I almost passed out sprinting after my dog."

History, on open questioning:
The patient has had palpitations on and off.
At times she would get chest pressure with exercise.
When she tried to run after her dog today, she felt lightheaded and almost fainted.

Symptoms and risk factors, if asked directly:
She gets short of breath after climbing 2 flights of steps.
She sometimes gets chest pressure that radiates up her neck when she exercises.
The chest pressure resolves with rest.
She does recall passing out after trying to run a marathon a few years ago. She thought she was dehydrated.
She sometimes feels her heart racing, usually during exertion.
She said her cholesterol was checked last year and she was told her numbers are fine.
She denies any swelling in her legs.
She does not wake up with shortness of breath in the middle of the night.

Duration of symptoms: The palpitations have been going on for years. She noticed the dyspnea on exertion about 8 months ago.

Activities of daily living: Able to perform ADLs, but exercise capacity is less compared to 2 years ago. She no longer goes on long hikes.

Family history:
Her mother and maternal aunt have reported palpitations and syncope.

Past Medical History:
None

Drug History:
Never smoked. Drinks a glass of wine when she goes out with friends about 2 glasses/month. No history of substance abuse.

Allergies:
Penicillin causes hives.

VIGNETTE 106
Osteitis deformans

Demographics:
Male, age 68

Presenting Complaint / Initial user input:
"My bones hurt."

History, on open questioning:
The patient has had low back pain and right knee pain for a number of years.
Now his left knee hurts.
The knee and back pain keep him up at night.

Symptoms and risk factors, if asked directly:
He says his right elbow and knees look deformed.
The knee pain is worse when he tries to walk.
He has been taking ibuprofen for a number of years to help manage the pain.
He has become "bow-legged" over the years
He does not have a history of cancer.
He does not have fevers or chills.
He is a retired mechanical engineer.

Duration of symptoms:
His symptoms have been ongoing for years.

Activities of daily living:
He can perform most ADLs on his own, but needs to take breaks if he needs to stand or walk for long periods.

Family history:
His father complained of back pain and pain in different joints. He ultimately died from osteosarcoma.

Past Medical History:
Right hip replacement, left knee replacement, arthritis of the knees, history of fracture of the left tibia, hearing loss in the right worse than the left.

Drug History:
10 pack-year history of tobacco smoking, quit 20 years ago; drinks a glass of wine most nights; marijuana use as a teenager

Allergies:
No known allergies.

<u>VIGNETTE 107</u>
Pulmonary embolism

Demographics:
-Female, Age 31

Presenting Complaint / Initial user input:
-“I’m having chest pain.”

History, on open questioning:
-The patient noticed sudden, sharp, right chest pain just after deplaning a flight from Australia.
-It was accompanied by shortness of breath, and a feeling of lightheadedness.
-Her chest pain is worse with taking a deep breath.
-She also feels like her “heart is racing”, and notes palpitations.
-No nausea or vomiting.

If asked directly:
-She has never had an episode like this before.
-She developed right calf pain halfway through the flight, and her ankle now looks swollen.
-Her pain does not radiate, but it “feels like it’s inside my chest.”
-Her chest wall does not hurt when she pushes on it.
-No recent cough, fever, chills, or recent illness.
-Patient is a salesperson and exercises daily without chest pain or shortness of breath, but today “now I can’t even walk” (i.e. *severe problems with performing daily activities).*

Family History:
-No relevant FH.

Past Medical History:
-PCOS
-Hormone replacement therapy
-Factor V Leiden

Social History:
-Active 1 pack/day smoker, no alcohol, no recreational drugs.

Allergies: NKDA

<u>VIGNETTE 108</u>
Syphilis male

Demographics:
Male, age 27

Presenting Complaint / Initial user input:
“I have a white ulcer on my penis.”

History, on open questioning:
The patient noticed a white lesion on the glans of his penis.
It doesn’t hurt, but has been there for about 1 week.
He also has a bump in his right groin area that he can feel when he is walking.
He has never had anything similar prior.

Symptoms and risk factors, if asked directly:
He denies having similar lesions elsewhere.
There is no discharge at the ulcer site.
He recalls no trauma there.
He had unprotected sex with a male partner about 3 weeks ago.
He denies having fevers or chills.
There is no discharge from the urethra.
He has a history of chlamydia infection which was treated about 6 months ago.
He denies fevers, chills, or weight loss.

Duration of symptoms:
The ulcer appeared about a week ago and has been unchanged during this time.

Activities of daily living:
He has no problems with ADLs.

Family history: Father with psoriasis

Past Medical History:
chlamydia infection.

Drug History:
No smoking history. Has mixed cocktails socially, up to 3 drinks a night. No history of substance abuse.

Allergies:
NKDA

<u>**VIGNETTE 109**</u>
Acute myocardial infarction

Demographics:
-Male, Age 55

Presenting Complaint / Initial user input:
-"I've just started having chest discomfort."

History, on open questioning:
-The discomfort started suddenly while shoveling snow.
-It is more of a pressure than a pain, "like an elephant sitting on my chest."
-Located in the center of the chest.
-Some associated pain in the neck and right shoulder.
-Something "doesn't feel right."

If asked directly:
-Also noting shortness of breath, nausea, sweating, and lightheadedness.
-Discomfort resolved some with sitting down.
-No sharp pains.
-No recent travel, calf pain, or lower extremity swelling.
-No recent cough, fever, or diarrhea.
-Has had increasing difficulty with exertion and ADLs (activities of daily living) over the last three months (i.e. *severe problems with performing daily activities)*
-Patient is a police officer, but has recently chosen to spend more time at his desk.

Family History:
-Father and paternal grandfather both "died of heart attacks."
-Mother his diabetes

Past Medical History:
-Diabetes (Type II)
-Hypertension

Social History:
-Active 1 pack/day smoker, 1-2 alcoholic drinks per day. No recreational drugs.

Allergies: NKDA
