# Supplementary material for: A Comparison of Artificial Intelligence and Human Doctors for the Purpose of Triage and Diagnosis
Source: Front Artif Intell. 2020 Nov 30;3:543405. doi: 10.3389/frai.2020.543405 (PMC7861270; doi:10.3389/frai.2020.543405)
Supplement: Supplementary file 2 [file datasheet2.pdf]

Supplementary Material

Table S1. Distribution of disease prior incidence rates, expressed in terms of person-years, from which disease priors in the model are calculated, aggregated over age and sex.

Incidence rate	% of Diseases
$\geq 10^{-1}$	2.0%
$10^{-2} - 10^{-1}$	5.9%
$10^{-3} - 10^{-2}$	19.4%
$10^{-4} - 10^{-3}$	46.3%
$10^{-5} - 10^{-4}$	23.3%
$10^{-6} - 10^{-5}$	2.2%
$< 10^{-6}$	0.8%

Table S2. Information about affiliations of role-play participants.

Coded name	Previously worked for Babylon	Provides GP consultations for Babylon	Involved in creation of clinical vignettes	Involved in development of Babylon AI products	Involved in development of Babylon Triage and Diagnostic Model
**Doctor A**	No	Yes	No	No	No
**Doctor B**	No	No	No	No	No
**Doctor C**	No	No	No	No	No
**Doctor D**	No	Yes	No	No	No
**Doctor E**	No	No	No	No	No
**Doctor F**	No	No	No	No	No
**Doctor G**	No	No	No	No	No
**Patient 1**	No	No	No	No	No
**Patient 2**	No	No	No	No	No
**Patient 3**	No	No	No	No	No
**Patient 4**	Yes	No	No	No	No
**Patient 5**	No	Yes	No	No	No
**Patient 6**	No	Yes	No	No	No
**Patient 7**	No	Yes	No	No	No
**Patient 8**	No	Yes	No	No	No
**Patient 9**	No	Yes	No	No	No
**Patient 10**	No	No	No	Yes	No

Table S3. Independent assessment of the quality of differential diagnosis by Judge-1. Each differential from the human doctors and the Babylon Triage and Diagnostic System (Babylon AI) was rated on a four point scale (poor/okay/good/excellent). The subjective quality of the Babylon Triage and Diagnostic System was found to be within the “Pass” range of values for human doctors.

	Poor	Okay	Good	Excellent	Pass	Cases
**Doctor A**	12.8%	25.5%	55.3%	6.4%	87.2%	47
**Doctor B**	21.8%	33.3%	37.2%	7.7%	78.2%	78
**Doctor C**	2.1%	41.7%	47.9%	8.3%	97.9%	48
**Doctor D**	7.8%	17.7%	62.8%	11.8%	92.2%	51
**Doctor E**	7.1%	5.7%	65.7%	21.4%	92.9%	70
**Doctor F**	5.9%	15.7%	74.5%	3.9%	94.1%	51
**Doctor G**	9.8%	43.1%	43.1%	3.9%	90.2%	51
**Doctor Average**	9.6%	26.1%	55.2%	9.1%	90.4%	56.6
**Babylon AI**	17.0%	34.0%	44.0%	5.0%	83.0%	100

Table S4. Independent assessment of the quality of differential diagnosis by Judge-2. Each differential from the human doctors and the Babylon Triage and Diagnostic System (Babylon AI) was rated on a four point scale (poor/okay/good/excellent). The subjective quality of the Babylon Triage and Diagnostic System was found to be outside of the “Pass” range of values for human doctors.

	Poor	Okay	Good	Excellent	Pass	Cases
**Doctor A**	12.8%	31.9%	34.0%	21.3%	87.2%	47
**Doctor B**	23.1%	28.2%	28.2%	20.5%	76.9%	78
**Doctor C**	6.3%	27.1%	47.9%	18.8%	93.8%	48
**Doctor D**	21.6%	23.5%	35.3%	19.6%	78.4%	51
**Doctor E**	18.6%	21.4%	42.9%	17.1%	81.4%	70
**Doctor F**	15.7%	23.5%	41.2%	19.6%	84.3%	51
**Doctor G**	13.7%	15.7%	41.2%	29.4%	86.3%	51
**Doctor Average**	16.0%	24.5%	38.7%	20.9%	84.0%	56.6
**Babylon AI**	48.0%	13.0%	27.0%	12.0%	52.0%	100

Table S5. Independent assessment of the quality of differential diagnosis by Judge-3. Each differential from the human doctors and the Babylon Triage and Diagnostic System (Babylon AI) was rated on a four point scale (poor/okay/good/excellent). The subjective quality of the Babylon Triage and Diagnostic System was found to be within the “Pass” range of values for human doctors.

	Poor	Okay	Good	Excellent	Pass	Cases
**Doctor A**	19.1%	29.8%	14.9%	36.2%	80.9%	47
**Doctor B**	48.7%	15.4%	9.0%	26.9%	51.3%	78
**Doctor C**	25.0%	16.7%	22.9%	35.4%	75.0%	48
**Doctor D**	33.3%	15.7%	23.5%	27.5%	66.7%	51
**Doctor E**	21.4%	20.0%	28.6%	30.0%	78.6%	70
**Doctor F**	37.2%	13.7%	21.6%	27.5%	62.8%	51
**Doctor G**	17.7%	11.8%	27.5%	43.1%	82.4%	51
**Doctor Average**	28.9%	17.6%	21.1%	32.4%	71.1%	56.6
**Babylon AI**	47.0%	11.0%	6.0%	36.0%	53.0%	100

Table S6. Diagnostic performance for all seven doctors and the Babylon Triage and Diagnostic System (Babylon AI), in terms of the recall (sensitivity), precision (positive predictive value) and F1 score (harmonic mean of precision and recall) against the disease modelled by the clinical vignette, after reweighting by the annual incidence of the disease modelled by the vignette.

	Average recall	Average precision	F1-score	Number of vignettes
**Doctor A**	52.0%	24.6%	33.4%	47
**Doctor B**	86.5%	37.2%	52.0%	78
**Doctor C**	99.96%	47.0%	64.0%	48
**Doctor D**	94.0%	33.6%	49.5%	51
**Doctor E**	96.3%	39.4%	55.9%	70
**Doctor F**	93.1%	50.2%	65.2%	51
**Doctor G**	75.1%	56.7%	64.6%	51
**Doctor average**	85.3%	41.2%	55.0%	56.6
**Babylon AI**	97.9%	83.3%	90.0%	100

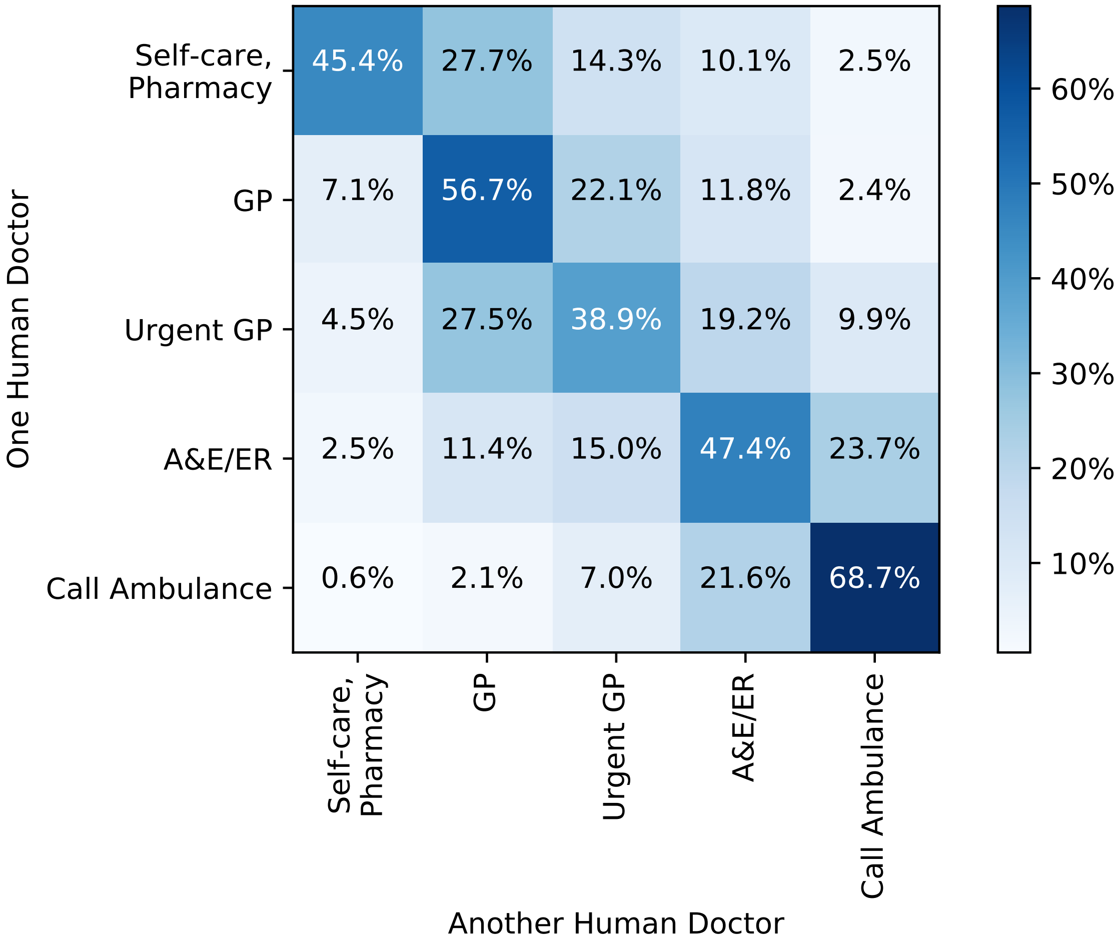

Figure S1. Confusion matrix between a single human doctor another human doctor (aggregated over all pairwise combinations of doctors). Considerable disagreement exists between the triage recommendations of different doctors, with confusion between all pairs of triage categories. Note that the *self-care* and *pharmacy* categories have been combined.

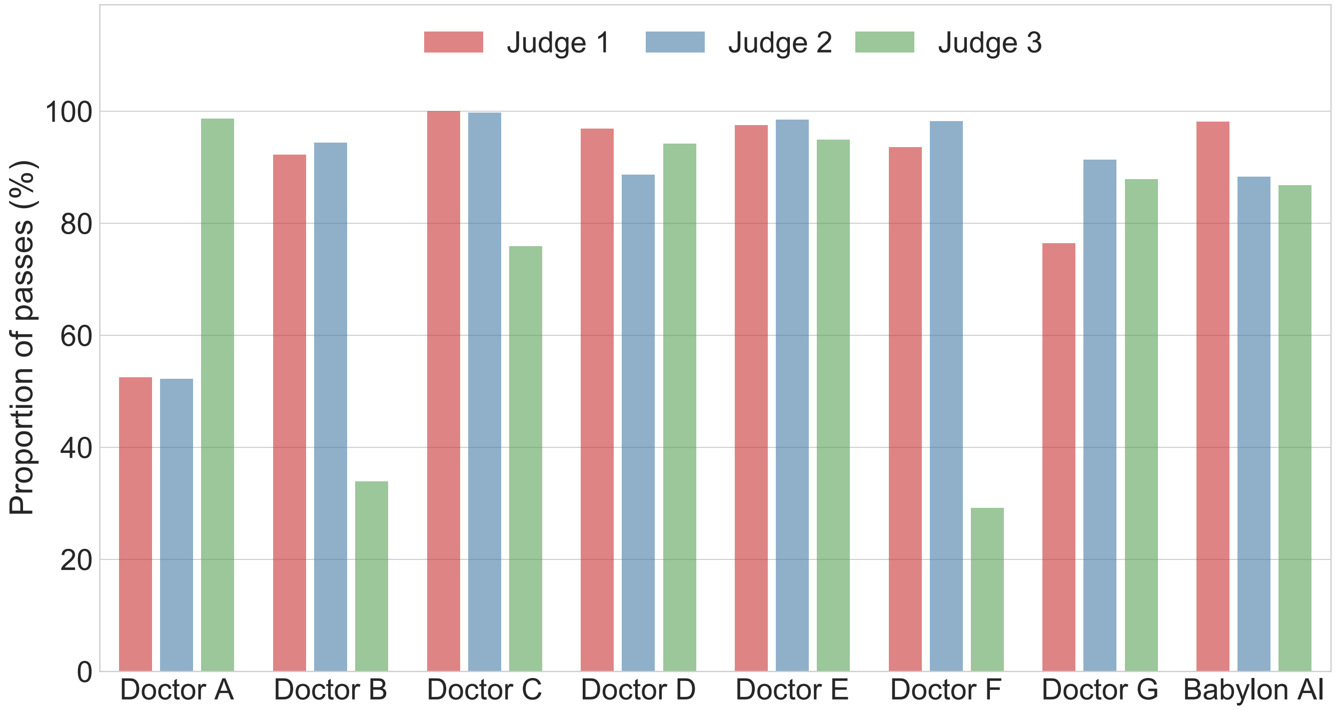

Figure S2. Percentage of differential diagnoses rated as "okay" or better by the judge and the two GPs for human doctors and the Babylon Triage and Diagnostic System (Babylon AI), after reweighting by the annual incidence of the disease modelled by the vignette.
